# Alzheimer's disease in patients prescribed statins: A real-world data analysis of U.S. patient health records

**DOI:** 10.1177/13872877261424220

**Published:** 2026-02-27

**Authors:** Daniel A. Novak, Najia Saleem, Paul C. Gerhardt, Drake Maestas, Nidhi Kejriwal, Elisa Vaezazizi, Ian Murray, Lama Al-Khoury

**Affiliations:** 1 Department of Social Medicine, Population, and Public Health, UC Riverside School of Medicine, Riverside, CA, USA; 2 UC Riverside School of Medicine, Riverside, CA, USA; 3 Department of Medical Education, 661705 Alice Walton School of Medicine, Bentonville, AR, USA; 4 Department of Psychiatry and Neuroscience, UC Riverside School of Medicine, Riverside, CA, USA

**Keywords:** Alzheimer's disease, cardiovascular risk factors, cholesterol, cohort study, drug repurposing, epidemiology, lipid metabolism, statins

## Abstract

**Background:**

Evidence from observational studies and randomized controlled trials (RCTs) remains discordant on the impact of statin therapy on long-term outcomes related to Alzheimer's disease. Observational studies find relatively large effect sizes; RCTs fail to demonstrate cognitive benefits. Methodological limitations in both approaches may explain the disconnect.

**Objective:**

To bridge the gap between observational and RCT studies, this study uses Real World Data (RWD) to evaluate the association between statin use and incident AD risk, and contributes additional detailed stratification by statin type and dosage.

**Methods:**

This observational analysis of EHR data from over 125 million U.S. patients through the TriNetX platform compared statin exposure in adults over 45 years old with a diagnosis of dyslipidemia, and no prior AD diagnosis, controlling for demographics, a range of known comorbidities, laboratory values, and medications. Primary outcomes were incident AD, other degenerative neurological diseases, and all-cause mortality. Sub-analyses compared risks by statin type and dosage.

**Results:**

Statin exposure was associated with a substantially lower risk of incident AD compared with non-exposure (RR = 0.69), similar to meta analyses of observational studies. Lipophilic and hydrophilic statins showed reduced AD risk compared to controls; hydrophilic statins showed a slightly greater protective effect. High-dose and low/medium-dose statins conferred similar risk reductions with no significant dose-dependent difference.

**Conclusions:**

Our findings provide evidence that statin therapy is associated with reduced risk of AD and related outcomes, extending prior observational data with broader population representation and rigorous confounder control. Pending further evidence, statins may be considered as part of comprehensive strategies to reduce AD risk.

## Introduction

Alzheimer's disease (AD) represents a significant and escalating public health crisis. As a progressive neurodegenerative disorder with no curative therapies, the identification of effective preventive strategies is a critical research priority. Statins (HMG-CoA reductase inhibitors), cornerstone therapies for cardiovascular disease prevention,^1^ have been a focus of investigation due to plausible neuroprotective mechanisms linked to cholesterol metabolism.^2–4^ Despite decades of research, the relationship between statin therapy and AD risk presents a number of inconsistent results.^5–6^ The literature is characterized by discordant findings stemming from fundamental methodological limitations in observational studies and randomized controlled trials (RCTs), creating an evidentiary challenge^7–10^ that this study seeks to address.

A substantial body of observational studies suggests a protective association between statin use and incident AD.^11–18^ Multiple large-scale meta-analyses of these studies have reported risk reductions for all-cause dementia and AD among statin users,^11,12,19^ with effect sizes ranging from 14% to 32%. For example, a 2022 meta-analysis of cohort and case-control studies by Olmastroni et al.^20^ reported an odds ratio (OR) of 0.68 for AD, while a 2025 umbrella review encompassing over seven million patients noted a hazard ratio (HR) of 0.82.^21^ These findings, however, are subject to methodological caveats that temper their interpretation.^22^ The most critical of these is the high potential for confounding by indication and residual confounding. Statins are prescribed to patients with cardiovascular risk factors (e.g., hyperlipidemia, hypertension), and these cardiovascular factors are themselves independent risk factors for dementia.^23^ Although statistical adjustments are employed, completely mitigating the confounding effects of these underlying conditions is difficult. Further, these studies are susceptible to selection biases, notably the “healthy user effect,” wherein individuals adherent to preventative medications like statins may also systematically engage in other health-promoting behaviors that independently lower dementia risk. Finally, meta-analyses in this domain often suffer from substantial heterogeneity, frequently aggregating data across different statin types (e.g., lipophilic versus hydrophilic), dosages, and treatment durations. This practice obscures crucial distinctions and prevents a granular analysis of whether specific statin properties or treatment regimens confer differential effects.

In contrast to observational data suggesting a protective association between statin use and incident dementia, randomized controlled trials have largely failed to demonstrate clear cognitive benefits, whether for primary prevention, prevention of progression from mild cognitive impairment (MCI) to dementia, or treatment of established AD.^24–28^ Primary prevention evidence from major statin RCTs and meta-analyses, including Ott et al. (2015) and a 2016 Cochrane review on prevention, have not provided convincing support that late-life initiation of statins prevents cognitive decline or incident dementia in cognitively normal older adults. These conclusions are consistent with more recent syntheses of observational cohort data, which suggest potential risk reduction but remain vulnerable to confounding and healthy-user bias.^5–7^

In secondary prevention situations (e.g., patients with MCI), statins have not been shown to reliably reduce progression to dementia, and dementia outcomes are often underpowered or ancillary within broader cardiovascular trials, as highlighted in prevention-focused reviews by McGuinness et al.^9^ and others. In contrast, Sano et al. 2011^27^ and the LEADe trial by Feldman et al.^28^ specifically evaluated simvastatin and atorvastatin, respectively, as treatment interventions in patients with mild to moderate AD and found no meaningful benefit on cognitive trajectories or global clinical outcomes.^26–30^ Recent treatment-focused meta-analyses, including Xuan et al.^26^ and the Cochrane review on statins for the treatment of dementia,^25^ similarly conclude that statin treatments do not exhibit clinically relevant symptomatic effect in established dementia. Large contemporary primary prevention trials such as STAREE^29^ were designed and powered primarily around cardiovascular endpoints, with cognitive decline and incident dementia included as secondary or exploratory outcomes, limiting inferences about statins as dedicated dementia-prevention agents. STAREE-related publications, including the STAREE-Mind Imaging Study, emphasize vascular and neuroimaging outcomes and illustrate how dementia endpoints are often embedded within broader cardiometabolic prevention frameworks rather than being the central focus of the trial design.

The current literature is thus defined by a conflict between promising but potentially biased observational findings and null results from RCTs not designed to test the hypothesis adequately in terms of endpoint or timeline. In addition to the overall impact of statins, the gaps in existing research leave critical clinical questions regarding 1) the impact of statin dosage, and 2) differences in impact between lipophilicity and hydrophilicity unanswered, as these data were not always germane to the original study designs. These issues may also contribute key variability to outcomes between observational and experimental outcomes.

To address these persistent methodological challenges and attempt to bridge the gap between findings in observational and prospective studies, this study leverages the TriNetX platform,^31^ a large-scale, federated network of real-world electronic health records. This approach capitalizes on the growing utility of real-world data in clinical research.^32–33^ Real-world data analyses can yield larger sample sizes that facilitate propensity score matching, and enable the construction of balanced cohorts and robust control for the confounding variables that have limited prior observational research. It also permits a granular analysis of comorbidities and a detailed exploration of outcomes across different statin subclasses and dosage levels. Finally, it allows us to examine multiple comorbidities and medications from across the user's health record, potentially reducing confounding through the elimination of additional confounders. By analyzing long-term outcomes in a diverse and representative patient population, this study may provide additional insights to inform the ongoing debate surrounding statin therapy and AD.

## Methods

This study examines whether, in routine clinical practice, statin therapy following dyslipidemia diagnosis is associated with subsequent incidence of AD and related outcomes among adults with disorders of lipoprotein metabolism, after adjustment for measured comorbid and contributing factors. Based on prior literature on lipid metabolism and neurodegeneration, we prespecified the following hypotheses:

- H0: There are no differences in AD incidence or related outcomes between statin-treated and untreated patients with dyslipidemia.
- H1: Patients receiving any statin at any dose at the index dyslipidemia event will have lower observed incidence of AD and related outcomes than those not prescribed statins.
- H2: The observed incidence of AD and related outcomes will differ across statin types (e.g., hydrophilic versus lipophilic agents).
- H3: Patients receiving higher-intensity statin regimens will show lower observed incidence of AD and related outcomes than those receiving lower-intensity regimens.

### Study design

Our design was informed by target trial emulation principles (explicit eligibility criteria, aligned time zero, pre-specified treatment strategies, and prospective follow-up), but we did not implement full causal g-methods or dynamic treatment strategies; therefore, our estimates should be interpreted as associations under residual confounding rather than as definitive causal effects.

To test our hypotheses and answer our study's questions, we conducted a retrospective cohort study using the TriNetX Analytics Platform on the United States (U.S.) Collaborative Network, a federated electronic health record (EHR) database comprising 72 healthcare organizations in the U.S., with a total patient pool of 126,379,149 on the final day of data collection (July 4, 2025). The U.S. Collaborative Network provides de-identified patient-level data (demographics, diagnoses, procedures, and medications) for real-world research, where structured data are imported directly from the EHRs of the partner organizations. The average length of patient records on TriNetX is approximately seven years and is censored at 20 years, or at the last patient fact. The 72 healthcare organizations in the network represent academic and private medical centers from each region in the US. Similarly, the dataset represents diversity of patients by ethnicity, language, and age. This study was determined to be non-human subjects research by the UC Riverside Institutional Review Board under DNHSR #30442. This study was reviewed using the STROBE checklist for observational study reporting to ensure consistency and quality.

### Statistical methods

All analyses were conducted on the TriNetX platform using its built-in cohort, propensity score, and outcomes tools. The TriNetX platform uses Java 11.0.16 (Apache Commons Math 3.6.1), R 4.0.2 (Hmisc 1-1, Survival 3.2-3), and Python 3.7 with the following packages: lifelines 0.22.4, matplotlib 3.5.1, numpy 1.21.5, pandas 1.3.5, scipy 1.7.3, and statsmodels 0.13.2 to conduct its analyses.

We first summarized baseline characteristics for each cohort at the index event (age at index, sex, calendar year, and all comorbidities and medication exposures). Standardized mean differences were used to assess balance before and after matching. Our primary exposure was statin therapy at any dose at the index event versus no recorded statin prescription in adults with dyslipidemia. Outcomes were defined a priori using ICD-10-CM codes and death flags: (1) incident AD (G30), (2) early-onset AD (EOAD, G30.0), (3) late-onset AD (LOAD, G30.1), (4) other degenerative neurological conditions (G31-G32), and (5) all-cause mortality (death flag or R99). For outcomes, follow-up time was measured in days from the index date to the first qualifying outcome code occurring after the index date; diagnoses of AD, EOAD, LOAD, or G31–G32 on or before the index date were treated as pre-existing and led to exclusion. For mortality, follow-up was measured from index to death, and deaths on the index date were counted as events.

### Measures of association analyses

For each outcome, we first estimated cumulative incidence and risk ratios (RRs) with 95% confidence intervals (CIs), and *p*-values for between-group differences were based on large-sample tests of proportions. All tests were two-sided with an alpha level of 0.05.

### Time-to-event analysis

We also conducted time-to-event analyses on the same matched cohorts. Kaplan-Meier curves were generated for AD, EOAD and LOAD, other degenerative neurological conditions, and all-cause mortality. Differences between curves were evaluated using log-rank tests, and hazard ratios (HRs) with 95% CIs were obtained from Cox proportional hazards models with exposure group as the sole covariate. The proportional hazards assumption was assessed using the platform's built-in test. We specifically inspected the Kaplan-Meier curves for dementia outcomes to evaluate for patterns suggestive of immortal time bias (e.g., prolonged early plateaus or abrupt late separation in the statin group); none were observed, consistent with our new-user design in which follow-up begins at the index date for both statin and non-statin cohorts.

### Time on treatment analysis

For patients in the statin cohort, we used TriNetX's *Time on Treatment* analytic to characterize real-world patterns of statin use. This module was used to estimate the time from the index dyslipidemia event to initiation of the first statin line of therapy (“time to treatment”) as well as the duration of that line (“time on treatment”), summarizing the distributions with the mean, standard deviation, median, and range.

### Error corrections

To account for multiple comparisons across the five prespecified outcomes within each analysis (AD, EOAD, LOAD, other degenerative neurological conditions, and all-cause mortality), we applied a Bonferroni correction (family-wise α = 0.05; α_B = 0.01 per outcome). All associations for AD and all-cause mortality with p < 0.001 remained statistically significant under this more stringent threshold. In contrast, several small associations, particularly for “other degenerative neurological conditions” (G31-G32) and dose- and subtype-specific comparisons, did not remain significant after correction.

### Cohort composition

We created two cohorts of adults aged 45 years or older with no prior diagnosis of AD at index. We selected this threshold to focus on age groups for whom statin prescriptions are common, and midlife cardiovascular risk is most relevant to later dementia risk. Cohort 1 included patients with disorders of lipoprotein metabolism and other lipidemias (E78) followed by documented statin therapy, and Cohort 2 included individuals with disorders of lipoprotein metabolism and other lipidemias (E78) but no history of statin therapy, serving as the comparison group. Initial cohort yields were large, reflecting our goal to assess real-world conditions. Statin users must have had one of the following statin medications following their diagnosis of a lipoprotein disorder: Fluvastatin (RxNorm: 41127), Rosuvastatin (RxNorm: 301542), Simvastatin (RxNorm: 36567), Atorvastatin (RxNorm: 83367), Pravastatin (RxNorm: 42463), Pitavastatin (RxNorm: 861634), Lovastatin (RxNorm: 6472).

Exclusion criteria for both cohorts corresponded with several known contributors to AD etiology or treatment that have been identified in contemporary research. These included:

- **Lifestyle and endocrine factors** such as type 2 diabetes (E11) or a recent HbA1c value over 7.0, nicotine dependence (F17; Z87.891) and alcohol-related disorders (F10), unspecified hypothyroidism (E03.9), and autoimmune thyroiditis (E06.3).
- **Other cognitive or neurological factors** such as unspecified psychosis (F29), Parkinson's disease (G20), epilepsy (G40), sleep disorders (G47), and Down Syndrome (Q90), other systemic atrophy primarily affecting CNS (G13.1), unspecified dementia (F03), cerebral infarction (I63). Patients were also excluded if their records included “Other degenerative neurological diseases” (G31 and G32) prior to the index date during the analysis.
- **Cardiovascular causes** like cerebrovascular diseases (I60-I69), complications and ill-defined descriptions of heart disease (I51), and vascular dementia (F01),
- **Infectious diseases** such as herpes or a history of herpes (Z86.19), prion and other viral diseases (A80-A89), and the herpes zoster vaccine (RxNorm: 1986821) to reduce odds of confounding, known herpes simplex infections (B00), and late syphilis (A52)
- **Common anti-AD drugs** like Donepezil (RxNorm: 135447), Rivastigmine (RxNorm: 183379), Galantamine (RxNorm: 4637), Memantine (RxNorm: 6719), Lecanemab (RxNorm: 2626143). Prescriptions recorded prior to or on the index date were treated as indicators of pre-existing cognitive impairment and led to exclusion. Prescriptions initiated after index were not used as exclusion criteria

Statin exposure was ascertained once at the index event and analyzed in an intention-to-treat framework; we did not time-update exposure during follow-up, so discontinuation or switching may lead to some exposure misclassification. However, our Time on Treatment analysis indicated that switching was a fairly rare event.

**Figure 1.**
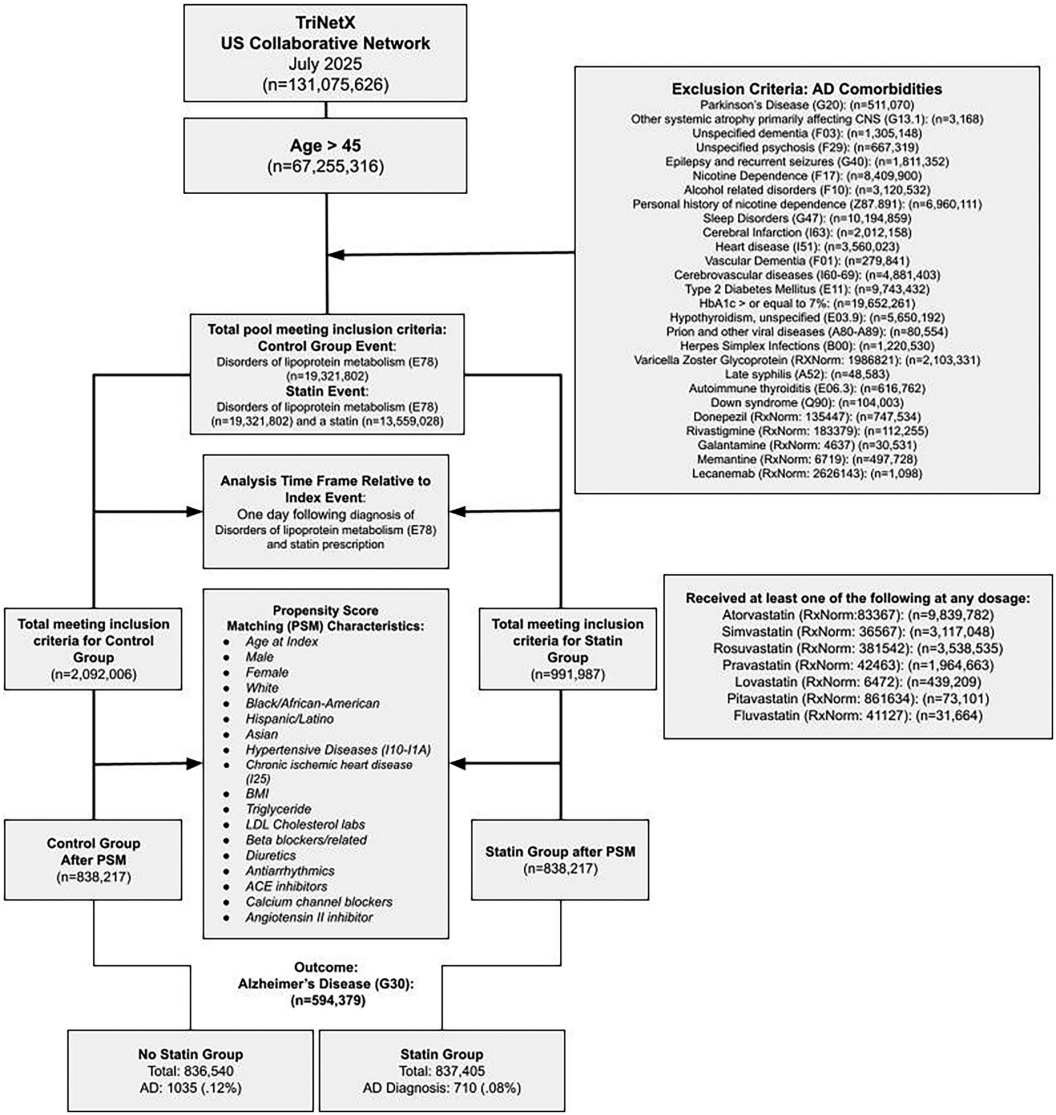
Cohort diagram for the study, illustrating the inclusion and exclusion criteria and the design of the cohorts and the patient counts at analysis runtime.

Our cohort diagram (Figure 1) illustrates the structure of the inclusion and exclusion criteria.

Type 2 diabetes and high HbA1c lab values are strongly associated with the development of dementia, and an indication for statin therapy in adults aged 40–75 years. As a result, the presence of diabetes in real-world data is tightly linked to statin prescribing, diabetes duration and severity, and use of additional therapies (insulin, GLP-1 agonists, SGLT2 inhibitors) that we cannot fully capture or time-align in this analysis. We therefore treated diabetes and markedly elevated HbA1c as major sources of confounding by indication and disease severity, and restricted the cohort to patients without type 2 diabetes or HbA1c ≥ 7% in both the statin and non-statin groups. This restriction removes diabetes as a competing causal pathway and avoids differential channeling of high-risk diabetic patients into the statin cohort, at the cost of reduced generalizability to patients with diabetes. Because the exclusion was applied symmetrically to both cohorts, it should not bias comparisons within the analytic sample. However, to ensure that diabetes did not serve as a confounder, we also present an analysis that does not exclude patients type 2 diabetic or high A1c, and a version that propensity score matches as well.

In contrast, hypertension is highly prevalent in middle-aged and older adults with dyslipidemia and is not, by itself, a categorical indication for statin therapy in the same way as diabetes. We therefore opted to retain patients with hypertensive diseases and adjust for hypertension using propensity score matching, rather than exclude a very large proportion of otherwise typical patients with lipid disorders.

To avoid immortal time bias in our analysis, we implemented a new-user design. For statin exposed patients, the index date was defined as the earliest date on which both a dyslipidemia diagnosis (E78) and a statin prescription were recorded. Follow-up began at statin initiation, and any time between first E78 and subsequent treatment initiation was not counted as exposed time. For non-users, the index date was the first recorded E78 diagnosis in patients with no statin prescriptions anywhere in their longitudinal record. Follow-up and outcome ascertainment began at these index dates for both groups. We also applied a washout period, excluding from the analysis any patient with a record of AD before the index date (or the first date wherein all conditions for cohort membership were met). No restrictions were placed on follow-up duration due to variance in age at index, and outcomes were captured from the index date until the end of available data (censored at the patient's last encounter in the network or at a maximum of 20 years of follow-up, whichever came first). Because the index date for statin users was defined as the earliest overlapping dyslipidemia diagnosis and statin prescription, whereas non-users were indexed at their first dyslipidemia diagnosis with no statin prescriptions in their record, small residual differences in time since initial dyslipidemia may remain between groups despite our efforts to align time zero.

### Propensity score matching

To address baseline imbalances and minimize confounding, we used propensity score matching to balance our cohorts along clinically relevant covariates related to AD risk and likelihood of statin treatment. These included age at index (represented as a continuous variable), sex (male/female), race and ethnicity (Black, White, Latino, and Asian), cardiovascular risk factors (e.g., hypertensive diseases, I10-I11A, chronic ischemic heart disease, I25), medications related to cardiovascular health (beta-blockers, diuretics, antiarrhythmics, ACE inhibitors, calcium channel blockers, and angiotensin II inhibitors). To control for a common confounder in existing research, we chose to propensity score match by common lab values in addition to diagnostic codes whenever possible. This included the most recent values for BMI (classified as normal/overweight at 15–30 and obese at 31–45 kg/m^2^), triglyceride level (classified as normal at 0–150 and high at 151–500 mg/dL), and LDL cholesterol (classified as normal at 0–100 or high at 101–500 mg/dL) on date of index. Propensity scores were used to perform 1:1 nearest-neighbor matching without replacement, using a caliper of 0.1 standard deviations of the logit of the propensity score. Matching was conducted after index and applying inclusion/exclusion criteria. Each statin user was matched to one non-user with a similar propensity score, resulting in two balanced cohorts for the main analysis (statins, n = 838,217). However, some were lost due to having been diagnosed with target outcomes (e.g., AD, non-AD degenerative neurological diseases) prior to their index event.

Before matching, statin users were older [mean age of 63.4 ± 11.8 versus 59.2 ± 12.8 years in controls, standardized mean difference (SMD): 0.34] and had a higher proportion of males (52.22% versus 45.40% in controls) and white individuals (71.46% versus 67.29%). Statin users also had a greater burden of cardiovascular comorbidities; for example, hypertension in 41.03% compared to 14.97% of controls (SMD 0.61). After PSM, baseline differences were significantly attenuated, with all standard mean difference values below the conventional value of 0.1. A complete list of covariates and their pre- and post-match scores is provided in Table 1, and a comprehensive list of all matching for sub-analysis cohorts in Supplemental Table 1. All outcome analyses were performed using these matched cohorts and matching criteria.

**Table 1.** Main analysis (statins versus control) cohort characteristics before and after propensity score matching.

		Before propensity score matching			after propensity score matching		
		Statins (%)	Control n (%)	SMD	Statins (%)	Control (%)	SMD
**Demographics**	**Identifier Code**						
Age at Index, mean (SD)	AI	63.36 (±11.81)	59.22 (±12.80)	0.34	62.34 (±11.62)	62.20 (±12.05)	0.011
Male	M	510,637 (52.22%)	916,547 (45.40%)	0.14	421,905 (50.33%)	421,278 (50.26%)	0.001
Female	F	467,042 (47.77%)	1,102,125 (54.59%)	0.14	416,228 (49.66%)	416,716 (49.72%)	0.001
White	2106–3	698,740 (71.46%)	1,358,662 (67.29%)	0.091	590,447 (70.44%)	592,899 (70.73%)	0.006
Black or African American	2054–5	101,195 (10.35%)	202,353 (10.02%)	0.011	87,854 (10.48%)	88,609 (10.57%)	0.003
Hispanic or Latino	2135–2	47,477 (4.79%)	131,321 (6.28%)	.070	43,246 (5.16%)	42,231 (5.04%)	0.006
Asian	2028–9	47,034 (4.74%)	110,144 (5.26%)	.027	41,387 (4.94%)	40,287 (4.81%)	0.006
**Diagnoses**	**ICD-10**						
Hypertensive diseases	I10-I1A	401,186 (41.03%)	302,206 (14.97%)	0.61	271,958 (32.45%)	270,694 (32.29%)	0.003
Chronic ischemic heart disease	I25	96,754 (9.89%)	33,557 (1.66%)	0.36	35,743 (4.26%)	33,257 (3.97%)	0.015
**Labs**	**LOINC**						
BMI (15–30 kg/m2)	9083	318,151 (32.54%)	535,597 (26.53%)	0.13	257,457 (30.71%)	259,132 (30.91%)	0.004
BMI (31–50 kg/m2)		146,251 (14.96%)	229,978 (11.39%)	0.11	117,170 (13.98%)	119,170 (14.22%)	0.007
LDL Cholesterol (0–100 mg/dL)	9002	134,433 (13.75%)	157,290 (7.79%)	0.19	95,506 (11.39%)	96,725 (11.54%)	0.005
LDL Cholesterol (101 + mg/dL)		340,879 (34.86%)	492,382 (24.39%)	0.23	274,051 (32.70%)	284,352 (33.92%)	0.026
Triglycerides (0–150 mg/dL)	9004	317,440 (32.47%)	446,843 (22.13%)	0.23	248,436 (29.64%)	256,080 (30.55%)	0.020
Triglycerides (151 + mg/dL)		179,330 (18.34%)	197,053 (9.76%)	0.25	131,793 (15.72%)	136,869 (16.33%)	0.017
**Medications**							
Antiarrythmics	CV300	155,800 (15.93%)	227,631 (11.27%)	0.14	120,852 (14.42%)	122,890 (14.66%)	0.007
Beta Blockers	CV100	124,755 (12.76%)	121,718 (6.03%)	0.23	83,512 (9.96%)	83,957 (10.02%)	0.002
Diuretics	CV700	112,389 (11.49%)	109,896 (5.44%)	0.22	78,923 (9.42%)	79,804 (9.52%)	0.004
ACE Inhibitors	CV800	86,975 (8.89%)	77,454 (3.84%)	0.21	59,863 (7.14%)	60,718 (7.24%)	0.004
Calcium Channel Blockers	CV200	89,861 (9.19%)	80,661 (4.00%)	0.21	61,258 (7.31%)	60,912 (7.27%)	0.002
Angiotensin II Inhibitors	CV805	71,880 (7.35%)	64,373 (3.19%)	0.19	49,401 (5.89%)	49,153 (5.86%)	0.001

### Outcome definitions

AD is the most common cause of dementia in older adults, but in clinical practice it often co-exists with, or is difficult to distinguish from, other neurodegenerative dementias. In this study we use AD (ICD-10 G30) as our *primary* outcome and refer to Alzheimer's disease and related dementias (ADRD) as a broader group of *secondary* outcomes that includes EOAD and LOAD (G30.0, G30.1) and other degenerative neurological conditions (G31-G32).

The primary analysis in this study focuses on incident AD, defined as a diagnosis of AD recorded after the index date. Patients with any AD diagnosis (ICD-10: G30) on or before the index date were considered to have pre-existing AD and were excluded from the analysis. In addition to overall AD incidence, we examined AD by subtype: LOAD (G30.1) and EOAD (G30.0) were evaluated as secondary outcomes. We also included a composite outcome of “Other degenerative neurological diseases,” defined by ICD-10 codes G31 and G32, to see if any observed effects were specific to AD compared to other dementia types (e.g., Lewy body dementia, frontotemporal dementia, etc.). Lastly, all-cause mortality was assessed as a key secondary outcome, given prior evidence that statin use may impact survival. Death was identified either from a recorded “deceased” indicator in the EHR or an associated mortality diagnosis code (R99, unspecified cause of mortality). Patients with any outcome code before the index were excluded from the analysis to ensure that all diagnoses or events were captured post-index. The selection of these codes is supported by other large-scale health records research studies.^15^

### Sub-analyses

In addition to our primary analysis comparing patients who used any statin with those who did not, we conducted two sub-analyses that may provide further insight into the relationship between statins and AD. The first sub-analysis focused on the type of statin prescribed, either lipophilic or hydrophilic, to determine whether one of these drug subgroups contributes more to differences in outcomes than the other. We compared lipophilic to non-statin users, hydrophilic to non-statin users, and lipophilic to hydrophilic statin users. The second sub-analysis focused on the dosages of statins (standard or high) to determine whether overall dosages impact patient outcomes. We again compared regular dosages of statins with non-statin users, high dosages of statins with non-statin users, and usual dosages with high-dosage users. The sub-analyses all focused on the same outcome definitions as in our primary analysis.

### Results

#### Time-on-treatment and follow-up time

To establish a baseline for exposure time, we conducted a time-on-treatment analysis of the statin group (n = 963,171) for the most common statins in the cohort. Patients on atorvastatin (n = 451,167) had a mean time-on-treatment of 1000.90 (SD = ± 1113.33), a median of 626 days, and a maximum of 7259 days. Patients on simvastatin (n = 136,229) had a mean time-on-treatment of 1461.13 (SD = ± 1519.09), a median of 947 days, and a maximum of 7211 days. Patients on lovastatin (n = 17,789) had a mean time-on-treatment of 1377.11 (SD = ± 1438.10), a median of 874 days, and a maximum of 7047 days. Patients on rosuvastatin (n = 184,311) had a mean time-on-treatment of 793.15 (SD = ± 933.51), a median of 478 days, and a maximum of 7078 days. Additionally, TriNetX was used to calculate the follow-up time as the mean, median, and interquartile range (IQR) for patients between index event and the last interaction with the health system in their record (up to a maximum of 20 years). In the matched cohorts, follow-up time in patient records was a mean of 1359.7 days (SD = 1420; median = 889 days; IQR = 1841) in statin-treated patients and 1217.7 (SD = 1340.3; median = 758 days; IQR = 1699) in non-statin patients.

#### Primary analysis: all statins and dosages

The primary analysis included two cohorts: A control cohort (n = 2,092,006) and a statin exposure cohort (n = 991,987). After propensity score matching, two balanced cohorts of 838,217 individuals each were included in the analysis. Following the index date of at least one calendar day after diagnosis of hyperlipidemia (and prescription of a statin) to remove individuals who were presenting with AD in their first visit, 710 individuals in the statin group and 1035 in the control group were diagnosed with AD, corresponding to a 31.5% reduction in risk (RR = 0.69; 95% CI, 0.62–0.75; p < 0.001). For EOAD (G30.0), 60 cases occurred in the statin group versus 98 in the control group (RR = 0.61; 95% CI, 0.44–0.84; p = 0.002). Late-onset AD (LOAD; G30.1) was diagnosed in 279 statin users and 391 controls (RR = 0.71; 95% CI, 0.61–0.83; p < 0.001). Conversely, statin use was associated with a slightly higher risk of other degenerative neurological conditions (G31, G32), with 3052 cases among statin users compared to 2514 in controls (RR = 1.21; 95% CI, 1.15–1.28; p < 0.001), and this association remained statistically significant after Bonferroni adjustment for multiple comparisons (adjusted p < 0.01). All-cause mortality was lower in statin users (30,854 deaths) compared to controls (35,300 deaths; RR = 0.87; 95% CI, 0.86–0.89; p < 0.001). These findings are summarized in Table 2.

**Table 2.** Outcomes for statins for the primary analysis of Alzheimer's disease, related outcomes, and all-cause mortality by statin group versus control.

Outcome	Cohort	Patients, N	Events, n (%)	Risk difference (95% CI)	*p*	Risk ratio (95% CI)
**Main Analysis: All Statins Versus Control**						
**Alzheimer's Disease (G30)**	Statin	837,407	710 (0.08)	−0.04% (−0.05% to −0.03%)	<0.001	0.69 (0.62 to 0.75)
	Control	836,540	1035 (0.12)			
**Early Onset AD (G30.0)**	Statin	838,158	60 (0.01)	−0.01% (−0.01% to −0.01%)	0.002	0.61 (0.44 to 0.84)
	Control	838,122	98 (0.02)			
**Late Onset AD (G30.1)**	Statin	838,045	279 (0.03)	−0.01% (−0.02% to −0.01%)	<0.001	0.71 (0.61 to 0.83)
	Control	837,840	391 (0.05)			
**Other Degenerative Neurological Conditions (G31, G32)**	Statin	836,276	3052 (0.36)	0.06% (0.05% to 0.08%)	<0.001	1.21 (1.15 to 1.28)
	Control	836,372	2514 (0.30)			
**Mortality (Deceased, R99)**	Statin	838,217	30,854 (3.68)	−0.53% (−0.59% to −0.47%)	<0.001	0.87 (0.86 to 0.89)
	Control	838,217	35,300 (4.21)			

In time-to-event analyses after propensity score matching and exclusion of patients with conditions prior to index, statin therapy was associated with a substantially lower hazard of incident AD. For overall AD (G30), 710 events occurred in the statin cohort and 1035 in controls, corresponding to a hazard ratio (HR) of 0.61 (95% CI 0.56–0.67; log-rank p < 0.001). For EOAD (G30.0), the HR was 0.55 (95% CI 0.40–0.76), and for LOAD (G30.1) the HR was 0.63 (95% CI 0.54–0.74), favoring statin therapy. In contrast, statin use was associated with a modestly higher hazard of other degenerative neurological conditions (G31-G32; HR 1.08, 95% CI 1.03–1.14), whereas all-cause mortality remained lower among statin users (HR 0.78, 95% CI 0.77–0.79).

**Figure 2.**
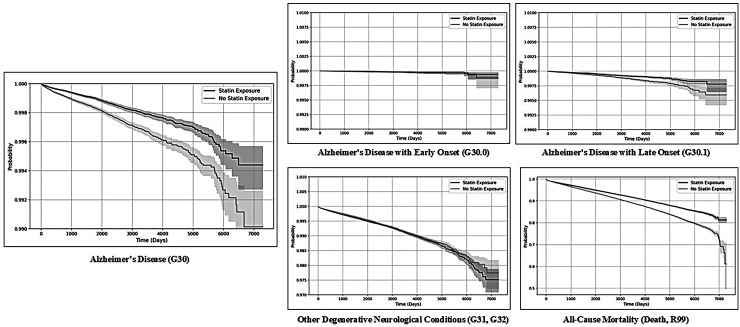
Kaplan-Meier curves for the outcomes in the primary analysis.

Kaplan-Meier curves for each outcome (Figure 2) show gradual, approximately proportional separation between the statin and non-statin cohorts over follow-up, with the statin curves consistently showing higher dementia-free survival. There is no prolonged early plateau in the statin group or abrupt late divergence that would suggest substantial immortal-time bias. This pattern is consistent with our cohort construction, which anchored time zero at the first dyslipidemia diagnosis with or without concurrent statin prescription and excluded individuals with dementia diagnoses prior to the index date. Taken together, the hazard ratios and accompanying Kaplan-Meier curves indicate a robust association between statin therapy and lower hazard of AD outcomes.

In a supplemental analysis, we treated all-cause mortality as a competing risk for incident AD using the TriNetX competing-risk analytic, which applies the Aalen-Johansen estimator to calculate cumulative incidence in the presence of death as a competing event. During follow-up there were 45,187 deaths and 1398 AD diagnoses; the cumulative incidence of AD remained low and was lower in statin users than non-users (0.14% versus 0.57%), despite statin users also experiencing substantially lower cumulative incidence of death (4.46% versus 22.61%). These findings indicate that the competing risk of death does not account for the observed association between statin therapy and reduced AD incidence.

### Sub-analysis 1: statin type

In a comparison between lipophilic statins (atorvastatin, simvastatin, pitavastatin, and lovastatin) and a matched control group, lipophilic statin use was associated with a significantly lower risk of AD (562 versus 794 cases; RR = 0.71; 95% CI, 0.64–0.79; p < 0.001). Similar reductions were observed for EOAD (43 versus 66 cases; RR = 0.65; 95% CI, 0.44–0.96; p = 0.028) and LOAD (250 versus 313 cases; RR = 0.80; 95% CI, 0.68–0.94; p = 0.008), though both outcomes have 95% CIs that approach 1. However, lipophilic statin use was linked to a higher risk of other degenerative neurological conditions (2239 versus 1847 cases; RR = 1.21; 95% CI, 1.14–1.29; p < 0.001). A lower risk of all-cause mortality was also observed (25,733 versus 27,110 deaths; RR = 0.95; 95% CI, 0.93–0.96; p < 0.001).

In the analysis of hydrophilic statins (pravastatin and rosuvastatin) versus controls, hydrophilic statin use was linked to a lower risk of AD (253 versus 390 cases; RR = 0.65; 95% CI, 0.55–0.76; p < 0.001), LOAD (78 versus 148 cases; RR = 0.53; 95% CI, 0.40–0.69; p < 0.001), and all-cause mortality (9866 versus 14,474 deaths; RR = 0.68; 95% CI, 0.67–0.70; p < 0.001). EOAD incidence was not statistically different between groups (26 versus 25 cases; RR = 1.04; 95% CI, 0.60–1.80; p = 0.889). Hydrophilic statin users also had similar or slightly higher rates of other degenerative neurological conditions (1106 versus 1016 cases; RR = 1.09; 95% CI, 1.00–1.19; p = 0.050).

A direct comparison between approximately 356,000 matched lipophilic and hydrophilic statin users found a higher risk of AD with lipophilic statins (332 versus 274 cases; RR = 1.21; 95% CI, 1.03–1.42; p = 0.018). The excess risk was more pronounced for LOAD (153 versus 84 cases; RR = 1.82; 95% CI, 1.40–2.38; p < 0.001), whereas EOAD incidence was not statistically significant between groups (30 versus 28 cases; RR = 1.07; 95% CI, 0.64–1.79; p = 0.793). The risk of other degenerative neurological conditions (1417 versus 1185 cases; RR = 1.20; 95% CI, 1.11–1.29; p < 0.001) and all-cause mortality (16,074 versus 11,311 deaths; RR = 1.42; 95% CI, 1.39–1.46; p < 0.001) were both higher among lipophilic statin users.

### Sub-analysis 2: statin dosage

In a comparison of low/medium-dose statins as defined by the American Heart Association (pravastatin, 10–20 mg; pitavastatin, 1–2 mg; simvastatin, 5–40 mg; rosuvastatin, 2.5–10 mg; fluvastatin, 20–40 mg; atorvastatin, 5–20 mg; lovastatin, 10–20 mg) versus controls, statin use was associated with a lower risk of AD (487 versus 623 cases; RR = 0.78; 95% CI, 0.69–0.88; p < 0.001) and all-cause mortality (18,400 versus 21,008 deaths; RR = 0.88; 95% CI, 0.86–0.89; p < 0.001). Risks of EOAD (44 versus 55 cases; RR = 0.80; 95% CI, 0.54–1.19; p = 0.27) and LOAD (203 versus 231 cases; RR = 0.88; 95% CI, 0.73–1.06; p = 0.18) were numerically lower but not statistically significant. Low/medium-dose statin use was associated with a higher risk of other degenerative neurological conditions (2107 versus 1563 cases; RR = 1.35; 95% CI, 1.26–1.44; p < 0.001).

Among high-dose statin (pravastatin, 40–90 mg; pitavastatin, 4 mg; simvastatin, 80 mg; rosuvastatin, 20–40 mg; fluvastatin, 80 mg; atorvastatin, 40–80 mg; lovastatin, 60 mg) users compared to controls, high-dose use was associated with reduced risk of AD (213 versus 288 cases; RR = 0.74; 95% CI, 0.62–0.88; p = 0.001), LOAD (85 versus 116 cases; RR = 0.73; 95% CI, 0.55–0.97; p = 0.03), and all-cause mortality (9457 versus 11,747 deaths; RR = 0.81; 95% CI, 0.78–0.83; p < 0.001). EOAD events were rare and similar between groups (20 versus 21 cases; RR = 0.95; 95% CI, 0.52–1.76; p = 0.88). High-dose statin use was also associated with a higher risk of other degenerative neurological conditions (894 versus 729 cases; RR = 1.23; 95% CI, 1.11–1.35; p < 0.001).

A comparison of matched low/medium-dose and high-dose users found no statistically significant differences in the risk of AD (267 versus 230 cases; RR = 1.16; 95% CI, 0.97–1.39; p = 0.10), EOAD (20 versus 21 cases; RR = 0.95; 95% CI, 0.52–1.76; p = 0.88), or LOAD (111 versus 91 cases; RR = 1.22; 95% CI, 0.93–1.61; p = 0.16). Low/medium-dose users had a modestly higher risk of other degenerative neurological conditions (1125 versus 972 cases; RR = 1.16; 95% CI, 1.06–1.26; p = 0.001) and higher all-cause mortality (11,926 versus 10,924 deaths; RR = 1.09; 95% CI, 1.06–1.12; p < 0.001) compared with high-dose users.

### Summary

**Figure 3.**
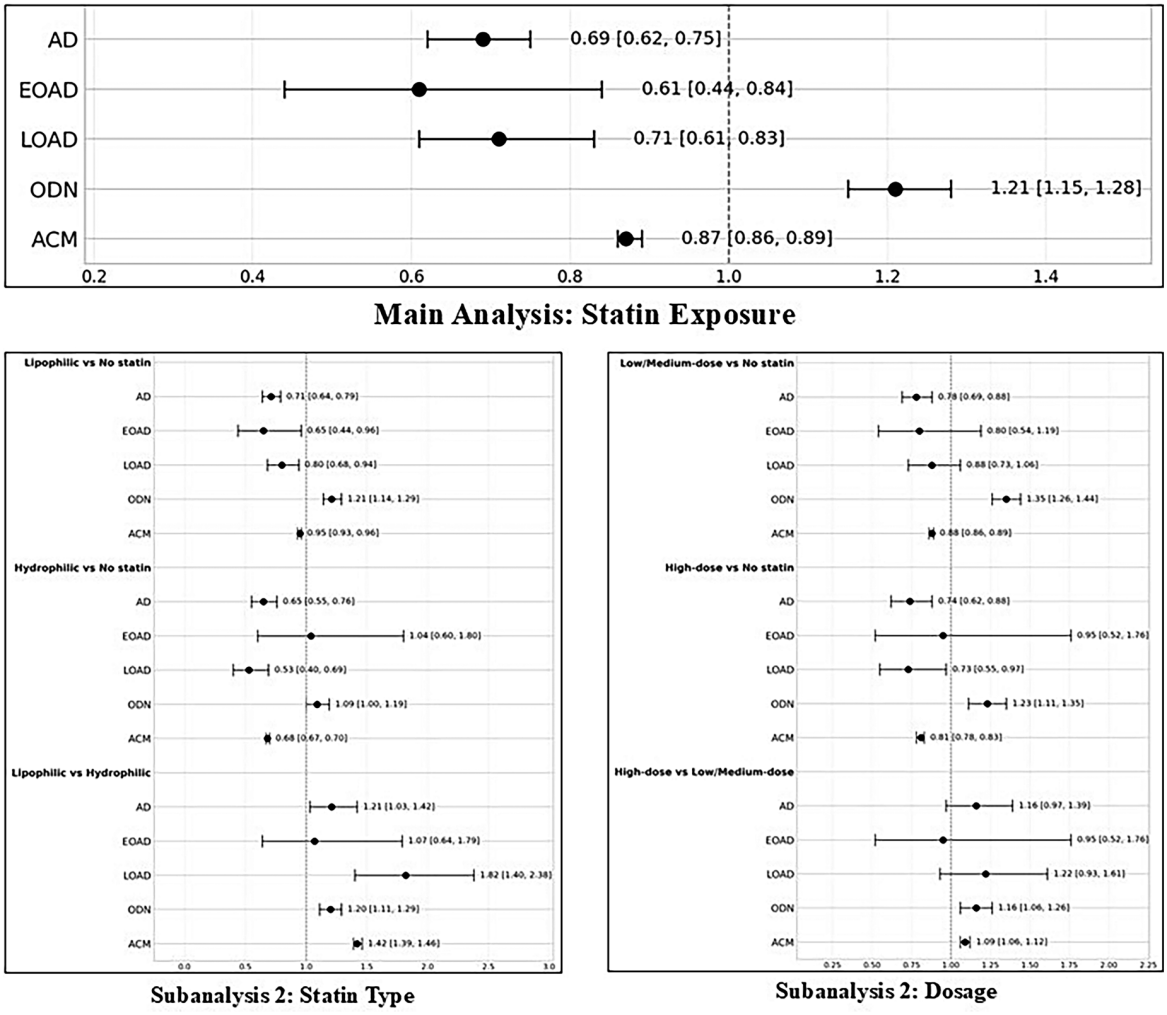
A forest plot illustrating the outcomes across each subanalysis and comparison.

The findings from our main analysis and two subanalyses are represented in Figure 3, a forest plot summarizing all of the outcomes from each set of contrasts.

### Sensitivity analysis

*Time-to-diagnosis.* Because dementia develops slowly and prescribing patterns may change in the setting of frailty or emerging cognitive impairment (e.g., deprescribing), we repeated the primary statin-versus-control comparison using a lagged outcome window. In this sensitivity analysis, AD and related outcomes were only ascertained beginning 3 years (1095 days) after the index date, and individuals who received a diagnosis of AD, other degenerative neurological conditions, or died during this lag period were excluded. Under this more conservative specification, incident AD remained less frequent among statin users than in matched controls, although point estimates were attenuated compared with the primary analysis. Among patients free of AD through the three-year lag, 375 statin users and 465 controls developed AD over follow-up (RR = 0.81; 95% CI, 0.70–0.92). Time-to-event analyses yielded a hazard ratio of 0.68 (95% CI, 0.59–0.78; log-rank p < 0.001), indicating a persistent 32% relative reduction in the hazard of AD among statin users even when early events were excluded. Patterns for AD subtypes were broadly consistent with the primary analysis. For EOAD, point estimates continued to favor statin users (RR = 0.91; 95% CI, 0.55–1.49; HR = 0.77; 95% CI, 0.47–1.26), but wide confidence intervals encompassed the null, reflecting the small number of events after imposing the three-year lag. For LOAD, the risk ratio was 0.87 (95% CI, 0.70–1.07), while the time-to-event analysis again showed a significant association (HR = 0.73; 95% CI, 0.59–0.90; log-rank p = 0.003). Taken together, these lagged analyses suggest that the lower AD risk observed in the primary analysis is not solely driven by short-term prescribing patterns or incipient dementia around the time of diagnosis. We also re-examined secondary outcomes using the three-year lag. The proportion of patients with other degenerative neurological diagnoses (G31-G32) was modestly higher in the statin group (0.2% versus 0.1%; RR = 1.20; 95% CI, 1.11–1.30), but Kaplan-Meier curves and hazard ratios were essentially null (HR = 1.01; 95% CI, 0.93–1.09), and absolute risk differences were extremely small. Statin therapy remained strongly associated with reduced all-cause mortality after the three-year lag (1.7% versus 1.9%; RR = 0.85; 95% CI, 0.83–0.87; HR = 0.72; 95% CI, 0.70–0.73; log-rank p < 0.001), consistent with established cardiovascular benefits. Because statin users experienced both lower mortality and lower or similar rates of AD despite having at least as much time at risk to develop dementia, these lagged analyses reinforce the robustness of the observed protective association.

*Type 2 diabetes.* Additionally, to examine whether our decision to exclude patients with type 2 diabetes or HbA1c ≥ 7.0 materially influenced the findings in our main analysis, we conducted a sensitivity analysis that retained these patients. In this analysis of matched cohorts (statin cohort n = 1,094,675; non-statin cohort n = 1,093,583, after excluding patients with AD prior to the risk window), the association between statin use and incident AD remained clearly protective but was attenuated relative to the primary analysis (RR = 0.71, 95% CI 0.66–0.77; HR = 0.62, 95% CI 0.57–0.68).

To further identify whether the inclusion of diabetic patients influenced the outcome, we repeated the propensity score matching process including type 2 diabetes diagnosis and hemoglobin A1c categories (3.5–6.9 and 7.0–13.0) as covariates, in order to more directly adjust for diabetes status and glycemic control among patients with available A1c measurements. When type 2 diabetes and hemoglobin A1c categories were explicitly included in the propensity score model and not excluded from the cohort, matched cohorts of 1,004,700 statin users and non-users yielded a similar association between statin therapy and incident AD (908 versus 1,22 AD cases; risk 0.09% versus 0.14%; RR = 0.64, 95% CI 0.59–0.69; p < 0.0001). These results are closely aligned with both our primary non-diabetic analysis and the diabetes-inclusive sensitivity analysis without explicit A1c adjustment, suggesting that confounding by diabetes status and glycemic control, as captured by diagnosis codes and A1c categories, does not materially account for the observed association although residual confounding by diabetes severity and treatment remains possible.

## Discussion

In this large, carefully matched real-world cohort, statin use was associated with lower recorded incidence of AD and lower all-cause mortality in non-diabetic adults with dyslipidemia. Our findings provide real-world evidence that statin therapy is associated with a reduced risk of AD (both EOAD and LOAD) and all-cause mortality. Using a large, propensity score matched cohort drawn from a diverse U.S. population and controlled for many common confounding comorbidities, we observed that statin users had a 31% lower risk of AD compared to non-users (RR = 0.69). This included consistent risk reductions across both EOAD (RR = 0.61) and LOAD (RR = 0.71) subtypes. Our primary analysis extends prior findings by leveraging a large, heterogeneous cohort, controlling for key cardiovascular and metabolic confounders, and stratifying results by statin type and dosage. This risk reduction is in line with the relative risk reductions reported in recent meta-analyses of observational studies (generally between 20% and 35%),^11,12,20,21^ and similar to Olmastroni et al.^20^ and their finding of an OR of .68, and much greater than the null or modest effects found in RCT-based studies and meta-analyses.

The magnitude of risk reduction observed in our study is clinically meaningful. While RCTs have generally failed to detect significant cognitive benefits from statins, this discrepancy may reflect differences in study design. By contrast, our analysis draws on long-term electronic health record data, enabling us to capture both cumulative statin exposure and the delayed onset of neurodegenerative conditions. Unlike other kinds of observational or retrospective studies (e.g., registries or purpose-built datasets), we were also able to access outcomes and events from across the patient's record within a health system and across multiple specialties. These methodological strengths may help explain why real-world data can reveal patterns that smaller, shorter RCTs have not.^20–21^^,28,30^

Our sub-analyses also leveraged electronic health record data to examine the complexity of the statin-AD relationship in ways that are often missing from current research. In comparisons with matched controls, both lipophilic (RR = 0.71; 95% CI, 0.64–0.79) and hydrophilic (RR = 0.65; 95% CI, 0.55–0.76) statins were associated with substantially lower AD risk relative to non-use. For LOAD, the associations were somewhat stronger for hydrophilic agents (RR = 0.53; 95% CI, 0.40–0.69) than for lipophilic agents (RR = 0.80; 95% CI, 0.68–0.94), whereas EOAD showed a protective association for lipophilic statins (RR = 0.65; 95% CI, 0.44–0.96) but no clear effect for hydrophilic statins (RR = 1.04; 95% CI, 0.60–1.80). In a direct head-to-head comparison, lipophilic statin users had higher risk of AD than hydrophilic statin users (RR = 1.21; 95% CI, 1.03–1.42), with an even larger excess for LOAD (RR = 1.82; 95% CI, 1.40–2.38). This pattern diverges from some prior meta-analyses that have reported more substantial effects for lipophilic statins, often attributed to higher blood-brain barrier penetration,^34,35^ but is broadly consistent with Poly et al.,^14^ who observed somewhat greater risk reduction among hydrophilic statin users. One plausible explanation is that lipophilic statins, while still associated with lower AD risk than non-use, may be preferentially prescribed to patients with heavier vascular comorbidity burdens, leading to residual confounding even after matching and exclusion. Conversely, hydrophilic agents such as rosuvastatin and pravastatin may be used in patients with different comorbidity profiles and may exert distinct pleiotropic or anti-inflammatory effects that translate into additional protection against neurodegeneration. Given the potential for confounding by indication and site-level prescribing patterns, these differences should be interpreted as hypothesis-generating rather than as definitive evidence to prefer one statin type over another for dementia prevention.

Additionally, our dosage-based analyses suggest that both low/medium-dose and high-dose statins are associated with lower AD risk compared with non-use, but do not show a clear dose-response gradient for dementia outcomes. Low/medium-dose statin use was associated with a modest reduction in AD risk (RR = 0.78; 95% CI, 0.69–0.88), and high-dose use showed a similar association (RR = 0.74; 95% CI, 0.62–0.88), while direct comparisons between low/medium- and high-dose users revealed no statistically significant differences in AD, EOAD, or LOAD risk. High-dose users, however, exhibited somewhat lower all-cause mortality than low/medium-dose users (RR = 1.09 for low/medium versus high dose), and both dose strata showed modestly higher rates of other degenerative neurological conditions (G31-G32) relative to controls. These patterns likely reflect a combination of clinical selection and residual confounding by indication, comorbidity burden, and intensity of cardiovascular risk management, rather than a simple causal gradient of cognitive benefit with higher statin doses. They are consistent with prior observational work, including a South Korean study in which treatment duration and dose were closely intertwined.^36^ Overall, our findings support the consensus view that, in late life, being on a statin at all, rather than the specific dose within the usual therapeutic range, is the more important factor for AD risk reduction. As before, our analyses primarily capture patients with one to five years of observed statin use and follow-up, and thus reflect late-life treatment of dyslipidemia in routine care, not lifetime primary prevention. These results should therefore be interpreted as evidence of late-life risk modification and should not be extrapolated to statin exposure initiated earlier in adulthood or maintained across the life course.

While our study excluded or matched for major cardiovascular events, it raises the possibility that improved survival among statin users may indirectly reduce AD risk by preventing vascular events and maintaining overall cerebrovascular health.^5,8,28^ This aligns with emerging evidence that vascular and metabolic risk factors such as hypertension, dyslipidemia, and diabetes are major contributors to cognitive decline and the specific pathophysiology of AD.^2–4^ By modifying these risk factors, statins may help preserve cognitive resilience over time.^14,20–21^ Similarly, other non-AD dementias (ICD-10 G31-G32) indicated increased association with risk of negative outcomes. In this study, the inclusion of non-AD dementias served as an exploratory, sensitivity-style test since we had no strong a priori expectation that statins would reduce these non-AD degenerative diagnoses, and we did not observe a consistent pattern of robust benefit or harm across all analyses. While future research is necessary, the lack of improved outcomes for G31 and G32 supports interpreting the statin findings as specific to AD rather than reflecting a generalized reduction in all neurologic diagnoses. Future research should examine how statins interact with these other non-AD dementia types to assess whether any benefits or harms are due to bias or to a physiological phenomenon.

### Clinical relevance

Our findings have several potential implications for clinical practice. First, they provide neurologists, cardiologists, and primary care providers with real-world evidence that may be used to counsel patients who are hesitant about statin therapy. While some patients discontinue statins due to concerns about cognitive side effects, these results suggest that statin users experience a substantially lower risk of AD as well as reduced all-cause mortality.^8,20^ These data may therefore be used to inform patients that, in addition to cardiovascular protection, statin therapy may also confer meaningful long-term neuroprotection. Second, the differential effects observed between hydrophilic and lipophilic statins may have implications for treatment selection in patients at heightened risk of dementia. Hydrophilic agents, such as rosuvastatin and pravastatin, were associated with lower AD risk and lower all-cause mortality when compared directly with lipophilic agents,^28,32^ suggesting that choice of statin type may warrant consideration when managing older adults with vascular risk who are also concerned about cognitive outcomes. Although lipophilic statins showed a protective association relative to non-use, their comparatively higher risks of other neurodegenerative diagnoses emphasize the importance of individualized prescribing. Third, our findings on dosage indicate that the cognitive benefit of statins is not dose-dependent beyond a certain threshold. Both low/medium- and high-dose statin users experienced reductions in AD risk, and there was no significant difference between these groups.^21^ This suggests that statin dosing may be optimized primarily according to cardiovascular indications, without the need for dose escalation to obtain additional neuroprotective effects.^7^

Taken together, these results support clinical arguments for prescribing statins beyond cholesterol and cardiovascular disease management alone. By situating statins within a broader framework of dementia prevention, our analysis supports their potential role as part of comprehensive risk-reduction strategies in aging populations.^20^ Tailoring statin choice to individual risk factors, emphasizing long-term adherence, and reframing statins as a therapy with dual cardiovascular and cognitive benefits may help clinicians improve uptake and adherence while also addressing patients’ growing concerns about dementia.

### Limitations

While our results are consistent with existing research and held up in the face of several additional sensitivity tests, they must be interpreted in light of several limitations. First, as an observational study, these analyses cannot establish causality, and residual confounding remains possible despite careful propensity score matching. Second, medication adherence and duration of statin use were not directly measured, meaning that some patients classified as statin users may have had intermittent or incomplete exposure. Third, our reliance on electronic health record diagnoses meant that undiagnosed, unreported, or misclassified cases of AD could not be captured. The absolute incidence of AD was low relative to population-based expectations, which could reflect under-ascertainment and misclassification in routine coding (e.g., as “other dementias”). This may exaggerate the apparent relative effect if under-coding is differential by statin status. Fourth, we were unable to identify cognitive testing data in TriNetX; these would provide additional details about degree of impairment for patients with AD. Similarly, we were not able identify individuals based on their apolipoprotein ε3 and ε4 gene variants due to limited and inconsistent availability of genetic information and laboratory values, though the relationship between these genes and statins are described in recent research.^37^ Fifth, our cohort reflects patients engaged with healthcare systems participating in TriNetX, which may not fully represent populations with limited access to care. Patient health information protections in the system also make it impossible to disaggregate by site, potentially introducing geographic variability. Sixth, although we implemented a new-user design and verified the absence of classic immortal-time patterns in Kaplan-Meier curves, some residual time-related bias (e.g., due to unobserved treatment delays or deprescribing) cannot be completely excluded. Seventh, our extensive exclusion criteria (including removing patients with prior cerebrovascular disease, vascular dementia, other neurodegenerative diagnoses, and anti-AD medications before index) were chosen to reduce reverse causation and competing dementia etiologies. However, they may have created artificially healthy populations that have lower risks of dementia than the average AD patient. Finally, despite extensive propensity score matching on demographics, cardiovascular risk factors, medications, and key laboratory values, residual confounding is possible. We could not adjust for education, socioeconomic status, detailed health behaviors, or cognitive reserve, and our data lack direct measures of adherence and preventive health behaviors. As a result, classic ‘healthy user’ and ‘healthy adherer’ effects remain plausible explanations for part of the observed associations. However, restricting on such factors can introduce selection or collider bias if the excluded conditions lie on, or are influenced by, pathways linking statin therapy and AD risk. This trade-off may contribute to the protective associations we observe and should be considered when interpreting our findings.

Taken together, the use of a new-user cohort anchored in routine care, exclusion of patients with diabetes and other high-risk conditions, extensive baseline restrictions, reliance on coded dementia diagnoses, and unmodeled clustering by site reduce some important sources of bias but may introduce others, including selection and collider bias and residual confounding by unmeasured factors. As such, our estimates should be interpreted as adjusted associations within a selected population of non-diabetic adults with dyslipidemia, rather than definitive causal effects generalizable to all statin-eligible patients.

### Future research

Future studies should focus on integrating genetic and biomarker data to identify patient subgroups most likely to benefit from statin therapy. Mendelian randomization studies and hybrid designs that combine real-world data with trial-like methodologies may help address residual confounding and provide a clearer understanding of causal pathways. Future randomized control trials of statin therapies should also include AD as an endpoint, with commensurately longer follow-up times. These data should also collect cognitive examination data that can be used to further enhance the validity of the findings. Additionally, studies exploring the timing of statin initiation, particularly whether midlife exposure provides greater neuroprotection than late-life initiation, are needed to inform clinical decision-making.

### Conclusion

In sum, our study contributes to a growing body of evidence that statins, beyond their established cardiovascular benefits, may reduce risk of AD. These findings support a broader consideration of statins as part of a multifaceted strategy for prevention of dementia, particularly in patients with elevated vascular risk. However, the translation of these observational findings into clinical guidelines will require continued investigation, including long-term, large-scale prospective studies designed to evaluate cognitive outcomes directly.

## Supplemental Material

sj-docx-1-alz-10.1177_13872877261424220 - Supplemental material for Alzheimer's disease in patients prescribed statins: A real-world data analysis of U.S. patient health records Supplemental material, sj-docx-1-alz-10.1177_13872877261424220 for Alzheimer's disease in patients prescribed statins: A real-world data analysis of U.S. patient health records by Daniel A. Novak, Najia Saleem, Paul C. Gerhardt, Drake Maestas, Nidhi Kejriwal, Elisa Vaezazizi, Ian Murray and Lama Al-Khoury in Journal of Alzheimer's Disease
