# Supplementary material for: Alzheimer's disease in patients prescribed statins: A real-world data analysis of U.S. patient health records
Source: J Alzheimers Dis. 2026 Feb 27;110(3):1474–89. doi: 10.1177/13872877261424220 (PMC13022028; doi:10.1177/13872877261424220)
Supplement: sj-docx-1-alz-10.1177_13872877261424220 - Supplemental material for Alzheimer's disease in patients prescribed statins: A real-world data analysis of U.S. patient health records [file sj-docx-1-alz-10.1177_13872877261424220.docx]

**Supplemental Material**

**Alzheimer’s disease in patients prescribed statins: A real-world data analysis of U.S. patient health records**

**Supplemental Table 1.** Complete propensity score matching results for all analyses.

|  | | **Before Propensity Score Matching** | | | | | | | **After Propensity Score Matching** | | | | | | | | |
| --- | --- | --- | --- | --- | --- | --- | --- | --- | --- | --- | --- | --- | --- | --- | --- | --- | --- |
|  |  | **Statins (%)** | | **Control n (%)** | | | | **SMD** | **Statins (%)** | | | **Control (%)** | | | **SMD** | | |
| **Main Analysis - All Statins versus Control** | | | | | | | | | | | | | | | | | |
| **Demographics** | **Identifier Code** | |  | |  | |  | | |  | | |  | | |  | |
| Age at Index, mean (SD) | AI | | 63.50 (11.74) | | 59.78 (12.84) | | 0.336 | | | 62.66 (11.60) | | | 62.62 (12.06) | | | 0.011 | |
| Male | M | | 510,637 (52.20%) | | 916,547 (45.40%) | | 0.137 | | | 421,905 (50.30%) | | | 421,278 (50.30%) | | | 0.001 | |
| Female | F | | 467,042 (47.80%) | | 1,102,125 (54.60%) | | 0.137 | | | 416,228 (49.70%) | | | 416,716 (49.70%) | | | 0.001 | |
| White | 2106-3 | | 698,740 (71.50%) | | 1,358,662 (67.30%) | | 0.091 | | | 590,447 (70.40%) | | | 592,899 (70.70%) | | | 0.006 | |
| Black or African American | 2054-5 | | 101,195 (10.30%) | | 202,353 (10.00%) | | 0.011 | | | 87,854 (10.50%) | | | 88,609 (10.60%) | | | 0.003 | |
| Hispanic or Latino | 2135-2 | | 47,477 (4.79%) | | 131,321 (6.28%) | | 0.070 | | | 43,246 (5.16%) | | | 42,231 (5.04%) | | | 0.006 | |
| Asian | 2028-9 | | 47,034 (4.74%) | | 110,144 (5.26%) | | 0.027 | | | 41,387 (4.94%) | | | 40,287 (4.81%) | | | 0.006 | |
| **Diagnoses** | **ICD-10** | |  | |  | |  | | |  | | |  | | |  | |
| Hypertensive diseases | I10-I1A | | 401,186 (41.00%) | | 302,206 (15.00%) | | 0.607 | | | 271,958 (32.40%) | | | 270,694 (32.30%) | | | 0.003 | |
| Chronic ischemic heart disease | I25 | | 96,754 (9.90%) | | 33,557 (1.70%) | | 0.358 | | | 35,743 (4.30%) | | | 33,257 (4.00%) | | | 0.015 | |
| **Labs** | **LOINC** | |  | |  | |  | | |  | | |  | | |  | |
| BMI (15-30 kg/m2) | 9083 | | 288,503 (30.76%) | | 467,916 (23.56%) | | 0.16 | | | 235,190 (28.67%) | | | 233,235 (28.43%) | | | 0.005 | |
| BMI (31-40 kg/m2) |  | | 132,091 (14.08%) | | 200,576 (10.10%) | | 0.12 | | | 106,000 (12.92%) | | | 106,480 (12.98%) | | | 0.001 | |
| LDL Cholesterol (0-100 mg/dL) | 9002 | | 118,515 (12.64%) | | 129,547 (6.52%) | | 0.21 | | | 83,772 (10.21%) | | | 83,875 (10.22%) | | | 0.001 | |
| LDL Cholesterol (101-500 mg/dL) |  | | 295,981 (31.56%) | | 413,549 (20.82%) | | 0.25 | | | 238,881 (29.12%) | | | 244,123 (29.76%) | | | 0.014 | |
| Triglycerides (0-150 mg/dL) | 9004 | | 317,440 (32.50%) | | 446,843 (22.10%) | | 0.234 | | | 248,436 (29.60%) | | | 256,080 (30.60%) | | | 0.020 | |
| Triglycerides (151-500 mg/dL) |  | | 179,330 (18.30%) | | 197,053 (9.80%) | | 0.249 | | | 131,793 (15.70%) | | | 136,869 (16.30%) | | | 0.017 | |
| **Medications** |  | |  | |  | |  | | |  | | |  | | |  | |
| Antiarrythmics | CV300 | | 155,800 (15.90%) | | 227,631 (11.30%) | | 0.136 | | | 120,852 (14.40%) | | | 122,890 (14.70%) | | | 0.007 | |
| Beta Blockers | CV100 | | 124,755 (12.80%) | | 121,718 (6.00%) | | 0.232 | | | 83,512 (10.00%) | | | 83,957 (10.00%) | | | 0.002 | |
| Diuretics | CV700 | | 112,389 (11.50%) | | 109,896 (5.40%) | | 0.219 | | | 78,923 (9.40%) | | | 79,804 (9.50%) | | | 0.004 | |
| ACE Inhibitors | CV800 | | 86,975 (8.90%) | | 77,454 (3.80%) | | 0.208 | | | 59,863 (7.10%) | | | 60,718 (7.20%) | | | 0.004 | |
| Calcium Channel Blockers | CV200 | | 89,861 (9.20%) | | 80,661 (4.00%) | | 0.211 | | | 61,258 (7.30%) | | | 60,912 (7.30%) | | | 0.002 | |
| Angiotensin II Inhibitors | CV805 | | 71,880 (7.40%) | | 64,373 (3.20%) | | 0.187 | | | 49,401 (5.90%) | | | 49,153 (5.90%) | | | 0.001 | |
| **Analysis 1A - Lipophilic Statin x Control** | | | | | | | | | | | | | | | | | |
| **Demographics** | | | | | | | | | | | | | | | | | |
| Age at Index | AI | 63.87 (11.91) | | | | 59.78 (12.84) | | 0.369 | | | 63.31 (11.78) | | | 63.35 (12.16) | | | 0.003 |
| White | 2106-3 | 443,974 (70.50%) | | | | 1,358,662 (67.30%) | | 0.069 | | | 407,902 (69.90%) | | | 412,273 (70.60%) | | | 0.016 |
| Male | M | 338,187 (53.70%) | | | | 916,547 (45.40%) | | 0.166 | | | 305,005 (52.20%) | | | 304,894 (52.20%) | | | <0.001 |
| Female | F | 291,804 (46.30%) | | | | 1,102,125 (54.60%) | | 0.166 | | | 278,748 (47.70%) | | | 278,756 (47.70%) | | | <0.001 |
| Black or African American | 2054-5 | 67,006 (10.60%) | | | | 202,353 (10.00%) | | 0.020 | | | 63,132 (10.80%) | | | 62,711 (10.70%) | | | 0.002 |
| Hispanic or Latino | 2135-2 | 35,145 (5.60%) | | | | 131,854 (6.50%) | | 0.040 | | | 33,331 (5.70%) | | | 32,289 (5.50%) | | | 0.008 |
| Asian | 2028-9 | 34,528 (5.50%) | | | | 118,610 (5.90%) | | 0.017 | | | 32,347 (5.50%) | | | 31,011 (5.30%) | | | 0.010 |
| **Diagnoses** | | | | | | | | | | | | | | | | | |
| Hypertensive diseases | I10-I1A | 263,261 (41.80%) | | | | 302,206 (15.00%) | | 0.623 | | | 218,865 (37.50%) | | | 217,827 (37.30%) | | | 0.004 |
| Chronic ischemic heart disease | I25 | 63,844 (10.10%) | | | | 33,557 (1.70%) | | 0.366 | | | 31,756 (5.40%) | | | 30,407 (5.20%) | | | 0.010 |
| **Labs** | | | | | | | | | | | | | | | | | |
| BMI (15-30 kg/m2) | 9083 | 183,420 (29.58%) | | | | 467,916 (23.55%) | | 0.14 | | | 165,205 (28.50%) | | | 164,444 (28.37%) | | | >0.001 |
| BMI (31-40 kg/m2) | 9083 | 85,323 (13.76%) | | | | 200,576 (10.10%) | | 0.11 | | | 76,857 (13.26%) | | | 76,661 (13.23%) | | | >0.001 |
| LDL Cholesterol (0-100 mg/dL) | 9002 | 80,841 (13.04%) | | | | 129,547 (6.52%) | | 0.22 | | | 65,864 (11.36%) | | | 65,277 (11.26%) | | | >0.001 |
| LDL Cholesterol (101-500 mg/dL) | 9002 | 179,128 (28.88%) | | | | 413,549 (20.82%) | | 0.19 | | | 164,054 (28.30%) | | | 166,227 (28.68%) | | | 0.01 |
| Triglycerides (0-150 mg/dL) | 9004 | 193,893 (30.80%) | | | | 446,843 (22.10%) | | 0.197 | | | 172,538 (29.60%) | | | 174,442 (29.90%) | | | 0.007 |
| Triglycerides (151-500 mg/dL) | 9004 | 108,990 (17.30%) | | | | 197,053 (9.80%) | | 0.222 | | | 94,366 (16.20%) | | | 98,677 (16.90%) | | | 0.020 |
| **Medications** | | | | | | | | | | | | | | | | | |
| Antiarrythmics | CV300 | 98,379 (15.60%) | | | | 227,631 (11.30%) | | 0.127 | | | 86,112 (14.70%) | | | 87,392 (15.00%) | | | 0.006 |
| Beta Blockers | CV100 | 81,149 (12.90%) | | | | 121,718 (6.00%) | | 0.236 | | | 64,970 (11.10%) | | | 64,749 (11.10%) | | | 0.001 |
| Diuretics | CV700 | 72,549 (11.50%) | | | | 109,896 (5.40%) | | 0.219 | | | 61,721 (10.60%) | | | 60,947 (10.40%) | | | 0.004 |
| ACE Inhibitors | CV800 | 57,105 (9.10%) | | | | 77,454 (3.80%) | | 0.214 | | | 48,394 (8.30%) | | | 48,084 (8.20%) | | | 0.002 |
| Calcium Channel Blockers | CV200 | 57,624 (9.10%) | | | | 80,661 (4.00%) | | 0.209 | | | 48,373 (8.30%) | | | 47,643 (8.20%) | | | 0.005 |
| Angiotensin II Inhibitors | CV805 | 43,349 (6.90%) | | | | 64,373 (3.20%) | | 0.169 | | | 37,377 (6.40%) | | | 36,718 (6.30%) | | | 0.005 |
| **Analysis 1B - Hydrophilic Statin x Control** | | | | | | | | | | | | | | | | | |
| **Demographics** | | | | | | | | | | | | | | | | | |
| Age at Index | AI | 63.37 (11.27) | | | | 59.78 (12.84) | | 0.332 | | | 62.99 (11.21) | | | 63.03 (11.79) | | | 0.003 |
| White | 2106-3 | 263,356 (73.00%) | | | | 1,358,662 (67.30%) | | 0.125 | | | 245,818 (72.40%) | | | 248,212 (73.10%) | | | 0.016 |
| Female | F | 180,646 (50.10%) | | | | 1,102,125 (54.60%) | | 0.091 | | | 173,020 (51.00%) | | | 174,527 (51.40%) | | | 0.009 |
| Male | M | 180,147 (49.90%) | | | | 916,547 (45.40%) | | 0.091 | | | 166,316 (49.00%) | | | 164,757 (48.50%) | | | 0.009 |
| Black or African American | 2054-5 | 35,513 (9.80%) | | | | 202,353 (10.00%) | | 0.006 | | | 34,063 (10.00%) | | | 34,326 (10.10%) | | | 0.003 |
| Hispanic or Latino | 2135-2 | 16,363 (4.50%) | | | | 131,854 (6.50%) | | 0.087 | | | 15,827 (4.70%) | | | 14,757 (4.30%) | | | 0.015 |
| Asian | 2028-9 | 16,857 (4.70%) | | | | 118,610 (5.90%) | | 0.054 | | | 16,205 (4.80%) | | | 15,587 (4.60%) | | | 0.009 |
| **Diagnoses** | | | | | | | | | | | | | | | | | |
| Hypertensive diseases | I10-I1A | 157,276 (43.60%) | | | | 302,206 (15.00%) | | 0.663 | | | 136,975 (40.40%) | | | 139,270 (41.00%) | | | 0.014 |
| Chronic ischemic heart disease | I25 | 45,270 (12.50%) | | | | 33,557 (1.70%) | | 0.434 | | | 27,712 (8.20%) | | | 25,053 (7.40%) | | | 0.029 |
| **Labs** | | | | | | | | | | | | | | | | | |
| BMI (15-30 kg/m2) | 9083 | 120,794 (36.48%) | | | | 467,916 (23.55%) | | 0.28 | | | 110,295 (35.26%) | | | 111,675 (35.71%) | | | 0.01 |
| BMI (31-40 kg/m2) | 9083 | 54,797 (16.55%) | | | | 200,576 (10.10%) | | 0.19 | | | 49,904 (15.96%) | | | 50,335 (16.09%) | | | 0.00 |
| LDL Cholesterol (0-100 mg/dL) | 9002 | 52,207 (15.77%) | | | | 129,547 (6.52%) | | 0.30 | | | 43,708 (13.97%) | | | 43,251 (13.83%) | | | 0.00 |
| LDL Cholesterol (101-500 mg/dL) | 9002 | 128,183 (38.72%) | | | | 413,549 (20.82%) | | 0.40 | | | 117,994 (37.73%) | | | 118,753 (37.97%) | | | 0.01 |
| Triglycerides (0-150 mg/dL) | 9004 | 137,762 (38.20%) | | | | 446,843 (22.10%) | | 0.355 | | | 124,640 (36.70%) | | | 125,535 (37.00%) | | | 0.005 |
| Triglycerides (151-500 mg/dL) | 9004 | 78,024 (21.60%) | | | | 197,053 (9.80%) | | 0.331 | | | 69,256 (20.40%) | | | 69,914 (20.60%) | | | 0.005 |
| **Medications** | | | | | | | | | | | | | | | | | |
| Antiarrythmics | CV300 | 75,469 (20.90%) | | | | 227,631 (11.30%) | | 0.265 | | | 65,707 (19.40%) | | | 66,669 (19.60%) | | | 0.007 |
| Beta Blockers | CV100 | 65,609 (18.20%) | | | | 121,718 (6.00%) | | 0.379 | | | 52,439 (15.50%) | | | 52,614 (15.50%) | | | 0.001 |
| Diuretics | CV700 | 55,600 (15.40%) | | | | 109,896 (5.40%) | | 0.331 | | | 47,356 (14.00%) | | | 47,731 (14.10%) | | | 0.003 |
| ACE Inhibitors | CV800 | 42,993 (11.90%) | | | | 77,454 (3.80%) | | 0.303 | | | 36,187 (10.70%) | | | 36,213 (10.70%) | | | <0.001 |
| Calcium Channel Blockers | CV200 | 47,953 (13.30%) | | | | 80,661 (4.00%) | | 0.335 | | | 39,325 (11.60%) | | | 39,139 (11.50%) | | | 0.002 |
| Angiotensin II Inhibitors | CV805 | 40,822 (11.30%) | | | | 64,373 (3.20%) | | 0.317 | | | 33,656 (9.90%) | | | 33,734 (9.90%) | | | 0.001 |
| **Analysis 1C - Lipophilic x Hydrophilic Statin** | | | | | | | | | | | | | | | | | |
| **Demographics** | | | | | | | | | | | | | | | | | |
| Age at Index | AI | 63.37 (11.27) | | | | 59.78 (12.84) | | 0.049 | | | 62.99 (11.21) | | | 63.03 (11.79) | | | 0.011 |
| White | 2106-3 | 443,974 (70.50%) | | | | 263,356 (73.00%) | | 0.056 | | | 260,066 (73.00%) | | | 259,821 (72.90%) | | | 0.002 |
| Male | M | 338,187 (53.70%) | | | | 180,147 (49.90%) | | 0.075 | | | 176,904 (49.60%) | | | 178,625 (50.10%) | | | 0.010 |
| Female | F | 291,804 (46.30%) | | | | 180,646 (50.10%) | | 0.075 | | | 179,513 (50.40%) | | | 177,786 (49.90%) | | | 0.010 |
| Black or African American | 2054-5 | 67,006 (10.60%) | | | | 35,513 (9.80%) | | 0.026 | | | 35,722 (10.00%) | | | 35,135 (9.90%) | | | 0.006 |
| Hispanic or Latino | 2135-2 | 35,145 (5.60%) | | | | 16,363 (4.50%) | | 0.048 | | | 15,216 (4.30%) | | | 16,307 (4.60%) | | | 0.015 |
| Asian | 2028-9 | 34,528 (5.50%) | | | | 16,857 (4.70%) | | 0.037 | | | 16,680 (4.70%) | | | 16,777 (4.70%) | | | 0.001 |
| **Diagnoses** | | | | | | | | | | | | | | | | | |
| Hypertensive diseases | I10-I1A | 263,261 (41.80%) | | | | 157,276 (43.60%) | | 0.036 | | | 152,755 (42.90%) | | | 153,846 (43.20%) | | | 0.006 |
| Chronic ischemic heart disease | I25 | 63,844 (10.10%) | | | | 45,270 (12.50%) | | 0.076 | | | 40,636 (11.40%) | | | 43,311 (12.20%) | | | 0.023 |
| **Labs** | | | | | | | | | | | | | | | | | |
| BMI (15-30 kg/m2) | 9083 | 183,420 (29.58%) | | | | 120,794 (36.48%) | | 0.15 | | | 119,461 (36.50%) | | | 118,226 (36.12%) | | | 0.01 |
| BMI (31-40 kg/m2) | 9083 | 85,323 (13.76%) | | | | 54,797 (16.55%) | | 0.08 | | | 53,668 (16.40%) | | | 53,479 (16.34%) | | | >0.001 |
| LDL Cholesterol (0-100 mg/dL) | 9002 | 80,841 (13.04%) | | | | 52,207 (15.77%) | | 0.08 | | | 47,546 (14.53%) | | | 50,689 (15.49%) | | | 0.03 |
| LDL Cholesterol (101-500 mg/dL) | 9002 | 179,128 (28.88%) | | | | 128,183 (38.72%) | | 0.21 | | | 126,610 (38.69%) | | | 125,105 (38.23%) | | | 0.01 |
| Triglycerides  (0-150 mg/dL) | 9004 | 193,893 (30.80%) | | | | 137,762 (38.20%) | | 0.156 | | | 133,758 (37.50%) | | | 134,485 (37.70%) | | | 0.004 |
| Triglycerides  (151-500 mg/dL) | 9004 | 108,990 (17.30%) | | | | 78,024 (21.60%) | | 0.109 | | | 75,680 (21.20%) | | | 76,096 (21.30%) | | | 0.003 |
| **Medications** | | | | | | | | | | | | | | | | | |
| Antiarrythmics | CV300 | 98,379 (15.60%) | | | | 75,469 (20.90%) | | 0.138 | | | 71,651 (20.10%) | | | 72,467 (20.30%) | | | 0.006 |
| Beta Blockers | CV100 | 81,149 (12.90%) | | | | 65,609 (18.20%) | | 0.147 | | | 61,537 (17.30%) | | | 62,267 (17.50%) | | | 0.005 |
| Diuretics | CV700 | 72,549 (11.50%) | | | | 55,600 (15.40%) | | 0.114 | | | 52,359 (14.70%) | | | 53,111 (14.90%) | | | 0.006 |
| ACE Inhibitors | CV200 | 57,624 (9.10%) | | | | 47,953 (13.30%) | | 0.132 | | | 44,132 (12.40%) | | | 45,079 (12.60%) | | | 0.008 |
| Calcium Channel Blockers | CV200 | 57,624 (9.10%) | | | | 47,953 (13.30%) | | 0.132 | | | 44,132 (12.40%) | | | 45,079 (12.60%) | | | 0.008 |
| Angiotensin II Inhibitors | CV805 | 43,349 (6.90%) | | | | 40,822 (11.30%) | | 0.155 | | | 37,323 (10.50%) | | | 37,719 (10.60%) | | | 0.004 |
| **Analysis 2A - Low Dose Statin x Control** | | | | | | | | | | | | | | | | | |
| **Demographics** | | | | | | | | | | | | | | | | | |
| Age at Index | AI | 63.39 (11.79) | | | | 59.78 (12.84) | | 0.307 | | | 62.94 (11.71) | | | 62.82 (12.11) | | | 0.022 |
| White | 2106-3 | 401,774 (73.50%) | | | | 1,358,662 (67.30%) | | 0.137 | | | 373,557 (72.80%) | | | 376,521 (73.40%) | | | 0.013 |
| Male | M | 270,958 (49.60%) | | | | 916,547 (45.40%) | | 0.084 | | | 250,794 (48.90%) | | | 249,534 (48.70%) | | | 0.005 |
| Female | F | 275,404 (50.40%) | | | | 1,102,125 (54.60%) | | 0.084 | | | 261,953 (51.10%) | | | 263,104 (51.30%) | | | 0.004 |
| Black or African American | 2054-5 | 51,856 (9.50%) | | | | 202,353 (10.00%) | | 0.018 | | | 49,867 (9.70%) | | | 49,477 (9.60%) | | | 0.003 |
| Hispanic or Latino | 2135-2 | 26,789 (4.90%) | | | | 131,854 (6.50%) | | 0.070 | | | 25,919 (5.10%) | | | 24,726 (4.80%) | | | 0.011 |
| Asian | 2028-9 | 32,325 (5.90%) | | | | 118,610 (5.90%) | | 0.002 | | | 30,439 (5.90%) | | | 29,654 (5.80%) | | | 0.007 |
| **Diagnoses** | | | | | | | | | | | | | | | | | |
| Hypertensive diseases | I10-I1A | 233,412 (42.70%) | | | | 302,206 (15.00%) | | 0.643 | | | 200,664 (39.10%) | | | 201,099 (39.20%) | | | 0.002 |
| Chronic ischemic heart disease | I25 | 44,102 (8.10%) | | | | 33,557 (1.70%) | | 0.301 | | | 27,977 (5.50%) | | | 25,375 (4.90%) | | | 0.023 |
| **Labs** | | | | | | | | | | | | | | | | | |
| BMI (15-30 kg/m2) | 9083 | 186,718 (33.79%) | | | | 467,916 (23.55%) | | 0.23 | | | 169,293 (32.56%) | | | 170,615 (32.81%) | | | 0.01 |
| BMI (31-40 kg/m2) | 9083 | 83,690 (15.14%) | | | | 200,576 (10.10%) | | 0.15 | | | 75,402 (14.50%) | | | 76,180 (14.65%) | | | >0.001 |
| LDL Cholesterol (0-100 mg/dL) | 9002 | 79,950 (14.47%) | | | | 129,547 (6.52%) | | 0.26 | | | 66,649 (12.82%) | | | 66,393 (12.77%) | | | >0.001 |
| LDL Cholesterol (101-500 mg/dL) | 9002 | 206,329 (37.34%) | | | | 413,549 (20.82%) | | 0.37 | | | 185,370 (35.65%) | | | 188,584 (36.27%) | | | 0.01 |
| Triglycerides (0-150 mg/dL) | 9004 | 213,370 (39.00%) | | | | 446,843 (22.10%) | | 0.374 | | | 190,257 (37.10%) | | | 192,702 (37.60%) | | | 0.010 |
| Triglycerides (151-500 mg/dL) | 9004 | 121,446 (22.20%) | | | | 197,053 (9.80%) | | 0.345 | | | 102,872 (20.10%) | | | 106,103 (20.70%) | | | 0.016 |
| **Medications** | | | | | | | | | | | | | | | | | |
| Antiarrythmics | CV300 | 100,466 (18.40%) | | | | 227,631 (11.30%) | | 0.201 | | | 88,742 (17.30%) | | | 90,419 (17.60%) | | | 0.009 |
| Beta Blockers | CV100 | 77,974 (14.30%) | | | | 121,718 (6.00%) | | 0.276 | | | 63,997 (12.50%) | | | 64,277 (12.50%) | | | 0.002 |
| Diuretics | CV700 | 72,245 (13.20%) | | | | 109,896 (5.40%) | | 0.270 | | | 61,002 (11.90%) | | | 60,981 (11.90%) | | | <0.001 |
| ACE Inhibitors | CV200 | 56,553 (10.40%) | | | | 80,661 (4.00%) | | 0.248 | | | 47,059 (9.20%) | | | 46,825 (9.10%) | | | 0.002 |
| Calcium Channel Blockers | CV200 | 56,553 (10.40%) | | | | 80,661 (4.00%) | | 0.248 | | | 47,059 (9.20%) | | | 46,825 (9.10%) | | | 0.002 |
| Angiotensin II Inhibitors | CV805 | 46,193 (8.50%) | | | | 64,373 (3.20%) | | 0.226 | | | 38,539 (7.50%) | | | 38,118 (7.40%) | | | 0.003 |
| **Analysis 2B - High Dose Statin x Control** | | | | | | | | | | | | | | | | | |
| **Demographics** | | | | | | | | | | | | | | | | | |
| Age at Index | AI | 64.12 (11.46) | | | | 59.78 (12.84) | | 0.388 | | | 63.58 (11.38) | | | 63.83 (11.91) | | | 0.015 |
| White | 2106-3 | 187,394 (73.40%) | | | | 1,358,662 (67.30%) | | 0.133 | | | 170,155 (72.60%) | | | 172,375 (73.60%) | | | 0.021 |
| Male | M | 151,431 (59.30%) | | | | 916,547 (45.40%) | | 0.281 | | | 134,963 (57.60%) | | | 135,293 (57.70%) | | | 0.003 |
| Female | F | 103,933 (40.70%) | | | | 1,102,125 (54.60%) | | 0.281 | | | 99,307 (42.40%) | | | 98,918 (42.20%) | | | 0.003 |
| Black or African American | 2054-5 | 28,468 (11.10%) | | | | 202,353 (10.00%) | | 0.037 | | | 26,770 (11.40%) | | | 26,933 (11.50%) | | | 0.002 |
| Hispanic or Latino | 2135-2 | 12,931 (5.10%) | | | | 131,854 (6.50%) | | 0.063 | | | 12,255 (5.20%) | | | 11,492 (4.90%) | | | 0.015 |
| Asian | 2028-9 | 11,769 (4.60%) | | | | 118,610 (5.90%) | | 0.057 | | | 11,009 (4.70%) | | | 10,324 (4.40%) | | | 0.014 |
| **Diagnoses** | | | | | | | | | | | | | | | | | |
| Hypertensive diseases | I10-I1A | 118,662 (46.50%) | | | | 302,206 (15.00%) | | 0.726 | | | 98,654 (42.10%) | | | 99,673 (42.50%) | | | 0.009 |
| Chronic ischemic heart disease | I25 | 48,528 (19.00%) | | | | 33,557 (1.70%) | | 0.594 | | | 27,968 (11.90%) | | | 26,220 (11.20%) | | | 0.023 |
| **Labs** | | | | | | | | | | | | | | | | | |
| BMI (15-30 kg/m2) | 9083 | 83,416 (31.40%) | | | | 467,916 (23.55%) | | 0.18 | | | 74,217 (30.27%) | | | 74,302 (30.31%) | | | >0.001 |
| BMI (31-40 kg/m2) | 9083 | 41,837 (15.75%) | | | | 200,576 (10.10%) | | 0.17 | | | 37,113 (15.14%) | | | 37,352 (15.23%) | | | >0.001 |
| LDL Cholesterol (0-100 mg/dL) | 9002 | 126.74 (49.53) | | | | 127.88 (34.29) | | 0.03 | | | 130.69 (49.29) | | | 120.64 (35.74) | | | 0.23 |
| LDL Cholesterol (101-500 mg/dL) | 9002 | 43,915 (16.53%) | | | | 129,547 (6.52%) | | 0.32 | | | 35,557 (14.50%) | | | 35,142 (14.33%) | | | >0.001 |
| Triglycerides  (0-150 mg/dL) | 9004 | 84,266 (33.00%) | | | | 446,843 (22.10%) | | 0.245 | | | 74,789 (31.90%) | | | 74,770 (31.90%) | | | <0.001 |
| Triglycerides  (151-500 mg/dL) | 9004 | 50,596 (19.80%) | | | | 197,053 (9.80%) | | 0.286 | | | 45,168 (19.30%) | | | 46,009 (19.60%) | | | 0.009 |
| **Medications** | | | | | | | | | | | | | | | | | |
| Antiarrythmics | CV300 | 50,066 (19.60%) | | | | 227,631 (11.30%) | | 0.232 | | | 42,813 (18.30%) | | | 43,439 (18.50%) | | | 0.007 |
| Beta Blockers | CV100 | 46,660 (18.30%) | | | | 121,718 (6.00%) | | 0.381 | | | 36,491 (15.60%) | | | 36,358 (15.50%) | | | 0.002 |
| Diuretics | CV700 | 36,818 (14.40%) | | | | 109,896 (5.40%) | | 0.303 | | | 31,310 (13.40%) | | | 31,537 (13.50%) | | | 0.003 |
| ACE Inhibitors | CV200 | 30,724 (12.00%) | | | | 80,661 (4.00%) | | 0.299 | | | 25,457 (10.90%) | | | 25,106 (10.70%) | | | 0.005 |
| Calcium Channel Blockers | CV200 | 30,724 (12.00%) | | | | 80,661 (4.00%) | | 0.299 | | | 25,457 (10.90%) | | | 25,106 (10.70%) | | | 0.005 |
| Angiotensin II Inhibitors | CV805 | 23,495 (9.20%) | | | | 64,373 (3.20%) | | 0.251 | | | 19,910 (8.50%) | | | 19,846 (8.50%) | | | 0.001 |
| **Analysis 2C - Low Dose Statin x High Dose Statin** | | | | | | | | | | | | | | | | | |
| **Demographics** | | | | | | | | | | | | | | | | | |
| Age at Index | AI | 63.39 (11.79) | | | | 64.12 (11.46) | | 0.080 | | | 64.13 (11.69) | | | 64.03 (11.46) | | | 0.012 |
| White | 2106-3 | 401,774 (73.50%) | | | | 187,394 (73.40%) | | 0.003 | | | 182,829 (73.70%) | | | 181,697 (73.20%) | | | 0.010 |
| Male | M | 270,958 (49.60%) | | | | 151,431 (59.30%) | | 0.196 | | | 145,268 (58.50%) | | | 144,775 (58.30%) | | | 0.004 |
| Female | F | 275,404 (50.40%) | | | | 103,933 (40.70%) | | 0.196 | | | 102,831 (41.40%) | | | 103,331 (41.60%) | | | 0.004 |
| Black or African American | 2054-5 | 51,856 (9.50%) | | | | 28,468 (11.10%) | | 0.054 | | | 27,816 (11.20%) | | | 27,709 (11.20%) | | | 0.001 |
| Hispanic or Latino | 2135-2 | 26,789 (4.90%) | | | | 12,931 (5.10%) | | 0.007 | | | 12,505 (5.00%) | | | 12,626 (5.10%) | | | 0.002 |
| Asian | 2028-9 | 32,325 (5.90%) | | | | 11,769 (4.60%) | | 0.059 | | | 11,184 (4.50%) | | | 11,624 (4.70%) | | | 0.008 |
| **Diagnoses** | | | | | | | | | | | | | | | | | |
| Hypertensive diseases | I10-I1A | 233,412 (42.70%) | | | | 118,662 (46.50%) | | 0.075 | | | 111,927 (45.10%) | | | 113,334 (45.70%) | | | 0.011 |
| Chronic ischemic heart disease | I25 | 44,102 (8.10%) | | | | 48,528 (19.00%) | | 0.324 | | | 41,070 (16.60%) | | | 41,267 (16.60%) | | | 0.002 |
| **Labs** | | | | | | | | | | | | | | | | | |
| BMI (15-30 kg/m2) | 9083 | 186,718 (33.79%) | | | | 83,416 (31.40%) | | 0.05 | | | 80,423 (31.10%) | | | 81,016 (31.33%) | | | >0.001 |
| BMI (31-40 kg/m2) | 9083 | 83,690 (15.14%) | | | | 41,837 (15.75%) | | 0.02 | | | 39,335 (15.21%) | | | 40,335 (15.60%) | | | 0.01 |
| LDL Cholesterol (0-100 mg/dL) | 9002 | 79,950 (14.47%) | | | | 43,915 (16.53%) | | 0.06 | | | 40,164 (15.53%) | | | 41,807 (16.17%) | | | 0.02 |
| LDL Cholesterol (101-500 mg/dL) | 9002 | 206,329 (37.34%) | | | | 79,846 (30.05%) | | 0.15 | | | 77,659 (30.03%) | | | 79,097 (30.58%) | | | 0.01 |
| Triglycerides  (0-150 mg/dL) | 9004 | 213,370 (39.00%) | | | | 84,266 (33.00%) | | 0.126 | | | 80,909 (32.60%) | | | 82,378 (33.20%) | | | 0.013 |
| Triglycerides  (151-500 mg/dL) | 9004 | 121,446 (22.20%) | | | | 50,596 (19.80%) | | 0.059 | | | 48,846 (19.70%) | | | 49,647 (20.00%) | | | 0.008 |
| **Medications** | | | | | | | | | | | | | | | | | |
| Antiarrythmics | CV300 | 100,466 (18.40%) | | | | 50,066 (19.60%) | | 0.031 | | | 47,280 (19.10%) | | | 48,061 (19.40%) | | | 0.008 |
| Beta Blockers | CV100 | 77,974 (14.30%) | | | | 46,660 (18.30%) | | 0.109 | | | 42,161 (17.00%) | | | 43,516 (17.50%) | | | 0.014 |
| Diuretics | CV700 | 72,245 (13.20%) | | | | 36,818 (14.40%) | | 0.035 | | | 33,845 (13.60%) | | | 35,292 (14.20%) | | | 0.017 |
| ACE Inhibitors | CV200 | 56,553 (10.40%) | | | | 30,724 (12.00%) | | 0.053 | | | 27,566 (11.10%) | | | 29,248 (11.80%) | | | 0.021 |
| Calcium Channel Blockers | CV200 | 56,553 (10.40%) | | | | 30,724 (12.00%) | | 0.053 | | | 27,566 (11.10%) | | | 29,248 (11.80%) | | | 0.021 |
| Angiotensin II Inhibitors | CV805 | 46,193 (8.50%) | | | | 23,495 (9.20%) | | 0.026 | | | 21,159 (8.50%) | | | 22,589 (9.10%) | | | 0.020 |

# **Supplemental Table 2.** Outcomes across all analyses

| **Outcome** | **Cohort** | **Patients, N (Post-PSM)** | **Events, n (%)** | **Risk Difference (95% CI)** | ***p*** | **Risk Ratio (95% CI)** |  |
| --- | --- | --- | --- | --- | --- | --- | --- |
| **Main Analysis: All Statins Versus Control** | | | | | | | |
| **Alzheimer’s Disease (G30)** | Statin | 837,405 | 710 (0.08) | -0.00% (-0.00% to -0.00%) | <0.001 | 0.69 (0.62 to 0.75) |  |
|  | Control | 836,540 | 1,035 (0.12) |  |  |  |  |
| **Early Onset AD (G30.0)** | Statin | 838,158 | 60 (0.01) | -0.00% (-0.00% to -0.00%) | 0.002 | 0.61 (0.44 to 0.84) |  |
|  | Control | 838,122 | 98 (0.01) |  |  |  |  |
| **Late Onset AD (G30.1)** | Statin | 838,045 | 279 (0.03) | -0.00% (-0.00% to -0.00%) | <0.001 | 0.71 (0.61 to 0.83) |  |
|  | Control | 837,840 | 391 (0.05) |  |  |  |  |
| **Other Degenerative Neurological Conditions (G31, G32)** | Statin | 836,276 | 3,052 (0.36) | 0.10% (0.00% to 0.10%) | <0.001 | 1.21 (1.15 to 1.28) |  |
|  | Control | 836,372 | 2,514 (0.30) |  |  |  |  |
| **Mortality (Deceased, R99)** | Statin | 838,217 | 30,854 (3.68) | -0.50% (-0.60% to -0.50%) | <0.001 | 0.87 (0.86 to 0.89) |  |
|  | Control | 838,217 | 35,300 (4.21) |  |  |  |  |
| **Analysis 1A: Lipophilic Statins Versus Control** | | | | | | | |
| **Alzheimer’s Disease (G30)** | Lipophilic Statin | 583,079 | 562 (0.10) | -0.00% (-0.10% to -0.00%) | <0.001 | 0.71 (0.64 to 0.79) | |
|  | Control | 582,535 | 794 (0.14) |  |  |  | |
| **Early Onset AD (G30.0)** | Lipophilic Statin | 583,766 | 43 (0.01) | -0.00% (-0.00% to -0.00%) | 0.028 | 0.65 (0.44 to 0.96) | |
|  | Control | 583,739 | 66 (0.01) |  |  |  | |
| **Late Onset AD (G30.1)** | Lipophilic Statin | 583,645 | 250 (0.04) | -0.00% (-0.00% to -0.00%) | 0.008 | 0.80 (0.68 to 0.94) | |
|  | Control | 583,504 | 313 (0.05) |  |  |  | |
| **Other Degenerative Neurological Conditions (G31, G32)** | Lipophilic Statin | 582,322 | 2,239 (0.38) | 0.10% (0.00% to 0.10%) | <0.001 | 1.21 (1.14 to 1.29) | |
|  | Control | 582,409 | 1,847 (0.32) |  |  |  | |
| **Mortality (Deceased, R99)** | Lipophilic Statin | 583,814 | 25,733 (4.41) | -0.20% (-0.30% to -0.20%) | <0.001 | 0.95 (0.93 to 0.96) | |
|  | Control | 583,814 | 27,110 (4.64) |  |  |  | |
| **Analysis 1B: Hydrophilic Statins Versus Control** | | | | | | | |
| **Alzheimer’s Disease (G30)** | Hydrophilic Statin | 339,154 | 253 (0.07) | -0.00% (-0.10% to -0.00%) | <0.001 | 0.65 (0.55 to 0.76) | |
|  | Control | 338,732 | 390 (0.12) |  |  |  | |
| **Early Onset AD (G30.0)** | Hydrophilic Statin | 339,358 | 26 (0.01) | 0.00% (-0.00% to 0.00%) | 0.889 | 1.04 (0.60 to 1.80) | |
|  | Control | 339,340 | 25 (0.01) |  |  |  | |
| **Late Onset AD (G30.1)** | Hydrophilic Statin | 339,334 | 78 (0.02) | -0.00% (-0.00% to -0.00%) | <0.001 | 0.53 (0.40 to 0.69) | |
|  | Control | 339,220 | 148 (0.04) |  |  |  | |
| **Other Degenerative Neurological Conditions (G31, G32)** | Hydrophilic Statin | 338,510 | 1,106 (0.33) | 0.00% (-0.00% to 0.10%) | 0.050 | 1.09 (1.00 to 1.19) | |
|  | Control | 338,524 | 1,016 (0.30) |  |  |  | |
| **Mortality (Deceased, R99)** | Hydrophilic Statin | 339,378 | 9,866 (2.91) | -1.40% (-1.40% to -1.30%) | <0.001 | 0.68 (0.67 to 0.70) | |
|  | Control | 339,378 | 14,474 (4.26) |  |  |  | |
| **Analysis 1C: Lipophilic Statins Versus Hydrophilic Statins** | | | | | | | |
| **Alzheimer’s Disease (G30)** | Lipophilic Statin | 356,063 | 332 (0.09) | 0.00% (0.00% to 0.00%) | 0.018 | 1.21 (1.03 to 1.42) | |
|  | Hydrophilic Statin | 356,218 | 274 (0.08) |  |  |  | |
| **Early Onset AD (G30.0)** | Lipophilic Statin | 356,427 | 30 (0.01) | 0.00% (-0.00% to 0.00%) | 0.793 | 1.07 (0.64 to 1.79) | |
|  | Hydrophilic Statin | 356,438 | 28 (0.01) |  |  |  | |
| **Late Onset AD (G30.1)** | Lipophilic Statin | 356,359 | 153 (0.04) | 0.00% (0.00% to 0.00%) | <0.001 | 1.82 (1.40 to 2.38) | |
|  | Hydrophilic Statin | 356,411 | 84 (0.02) |  |  |  | |
| **Other Degenerative Neurological Conditions (G31, G32)** | Lipophilic Statin | 355,497 | 1,417 (0.40) | 0.10% (0.00% to 0.10%) | <0.001 | 1.20 (1.11 to 1.29) | |
|  | Hydrophilic Statin | 355,506 | 1,185 (0.33) |  |  |  | |
| **Mortality (Deceased, R99)** | Lipophilic Statin | 356,457 | 16,074 (4.51) | 1.30% (1.20% to 1.40%) | <0.001 | 1.42 (1.39 to 1.46) | |
|  | Hydrophilic Statin | 356,457 | 11,311 (3.17) |  |  |  | |
| **Analysis 2A: Low/Medium Dosage Statins Versus Control** | | | | | | | |
| **Alzheimer’s Disease (G30)** | Low/Medium Statin | 512,233 | 487 (0.10) | -0.00% (-0.00% to -0.00%) | <0.001 | 0.78 (0.69 to 0.88) |  |
|  | Control | 511,812 | 623 (0.12) |  |  |  |  |
| **Early Onset AD (G30.0)** | Low/Medium Statin | 512,743 | 44 (0.01) | -0.00% (-0.00% to 0.00%) | 0.269 | 0.80 (0.54 to 1.19) |  |
|  | Control | 512,720 | 55 (0.01) |  |  |  |  |
| **Late Onset AD (G30.1)** | Low/Medium Statin | 512,661 | 203 (0.04) | -0.00% (-0.00% to 0.00%) | 0.178 | 0.88 (0.73 to 1.06) |  |
|  | Control | 512,542 | 231 (0.05) |  |  |  |  |
| **Other Degenerative Neurological Conditions (G31, G32)** | Low/Medium Statin | 511,524 | 2,107 (0.41) | 0.10% (0.10% to 0.10%) | <0.001 | 1.35 (1.26 to 1.44) |  |
|  | Control | 511,540 | 1,563 (0.31) |  |  |  |  |
| **Mortality (Deceased, R99)** | Low/Medium Statin | 512,786 | 18,400 (3.59) | -0.50% (-0.60% to -0.40%) | <0.001 | 0.88 (0.86 to 0.89) |  |
|  | Control | 512,786 | 21,008 (4.10) |  |  |  |  |
| **Analysis 2B: High Dosage Statins Versus Control** | | | | | | | |
| **Alzheimer’s Disease (G30)** | High Dose Statin | 234,064 | 213 (0.09) | -0.00% (-0.10% to -0.00%) | 0.001 | 0.74 (0.62 to 0.88) |  |
|  | Control | 233,798 | 288 (0.12) |  |  |  |  |
| **Early Onset AD (G30.0)** | High Dose Statin | 234,259 | 20 (0.01) | -0.00% (-0.00% to 0.00%) | 0.876 | 0.95 (0.52 to 1.76) |  |
|  | Control | 234,246 | 21 (0.01) |  |  |  |  |
| **Late Onset AD (G30.1)** | High Dose Statin | 234,224 | 85 (0.04) | -0.00% (-0.00% to -0.00%) | 0.029 | 0.73 (0.55 to 0.97) |  |
|  | Control | 234,164 | 116 (0.05) |  |  |  |  |
| **Other Degenerative Neurological Conditions (G31, G32)** | High Dose Statin | 233,622 | 894 (0.38) | 0.10% (0.00% to 0.10%) | <0.001 | 1.23 (1.11 to 1.35) |  |
|  | Control | 233,649 | 729 (0.31) |  |  |  |  |
| **Mortality (Deceased, R99)** | High Dose Statin | 234,281 | 9,457 (4.04) | -1.00% (-1.10% to -0.90%) | <0.001 | 0.81 (0.78 to 0.83) |  |
|  | Control | 234,281 | 11,747 (5.01) |  |  |  |  |
| **Analysis 2C: Low/Medium Dosage Statins Versus High Dosage Statins** | | | | | | |  |
| **Alzheimer’s Disease (G30)** | Low/Medium Statin | 247,814 | 267 (0.11) | 0.00% (-0.00% to 0.00%) | 0.096 | 1.16 (0.97 to 1.39) |  |
|  | High Dose Statin | 247,879 | 230 (0.09) |  |  |  |  |
| **Early Onset AD (G30.0)** | Low/Medium Statin | 248,108 | 20 (0.01) | -0.00% (-0.00% to 0.00%) | 0.876 | 0.95 (0.52 to 1.76) |  |
|  | High Dose Statinʰ | 248,101 | 21 (0.01) |  |  |  |  |
| **Late Onset AD (G30.1)** | Low/Medium Statin | 248,056 | 111 (0.04) | 0.00% (-0.00% to 0.00%) | 0.159 | 1.22 (0.93 to 1.61) |  |
|  | High Dose Statin | 248,057 | 91 (0.04) |  |  |  |  |
| **Other Degenerative Neurological Conditions (G31, G32)** | Low/Medium Statin | 247,459 | 1,125 (0.45) | 0.10% (0.00% to 0.10%) | 0.001 | 1.16 (1.06 to 1.26) |  |
|  | High Dose Statin | 247,389 | 972 (0.39) |  |  |  |  |
| **Mortality (Deceased, R99)** | Low/Medium Statin | 248,123 | 11,926 (4.81) | 0.40% (0.30% to 0.50%) | <0.001 | 1.09 (1.06 to 1.12) |  |
|  | High Dose Statin | 248,123 | 10,924 (4.40) |  |  |  |  |
